# Impaired Intestinal Akkermansia muciniphila and Aryl Hydrocarbon Receptor Ligands Contribute to Nonalcoholic Fatty Liver Disease in Mice

**DOI:** 10.1128/mSystems.00985-20

**Published:** 2021-02-23

**Authors:** Zunji Shi, Hehua Lei, Gui Chen, Peihong Yuan, Zheng Cao, Hooi-Leng Ser, Xuehang Zhu, Fang Wu, Caixiang Liu, Manyuan Dong, Yuchen Song, Yangyang Guo, Chuan Chen, Kexin Hu, Yifan Zhu, Xin-an Zeng, Jinlin Zhou, Yujing Lu, Andrew D. Patterson, Limin Zhang

**Affiliations:** a Chinese Academy of Sciences (CAS) Key Laboratory of Magnetic Resonance in Biological Systems, State Key Laboratory of Magnetic Resonance and Atomic and Molecular Physics, National Centre for Magnetic Resonance in Wuhan, Innovation Academy for Precision Measurement Science and Technology, CAS, Wuhan, China; b School of Food Science and Engineering, South China University of Technology, Guangzhou, China; c School of Biomedical and Pharmaceutical Sciences, Guangdong University of Technology, Guangzhou, China; d University of the Chinese Academy of Sciences, Beijing, China; e Engineering Research Academy of High Value Utilization of Green Plants, Meizhou, China; f Center for Molecular Toxicology and Carcinogenesis, Department of Veterinary and Biomedical Sciences, Pennsylvania State University, Pennsylvania, USA; g Wuhan National Laboratory for Optoelectronics, Wuhan, China; Dalhousie University

**Keywords:** microbiome, gut-liver axis, *Akkermansia muciniphila*, AHR ligands

## Abstract

Noncaloric artificial sweeteners (NAS) are extensively introduced into commonly consumed drinks and foods worldwide. However, data on the health effects of NAS consumption remain elusive. Saccharin and sucralose have been shown to pass through the human gastrointestinal tract without undergoing absorption and metabolism and directly encounter the gut microbiota community. Here, we aimed to identify a novel mechanism linking intestinal Akkermansia muciniphila and the aryl hydrocarbon receptor (AHR) to saccharin/sucralose-induced nonalcoholic fatty liver disease (NAFLD) in mice. Saccharin/sucralose consumption altered the gut microbial community structure, with significant depletion of A. muciniphila abundance in the cecal contents of mice, resulting in disruption of intestinal permeability and a high level of serum lipopolysaccharide, which likely contributed to systemic inflammation and caused NAFLD in mice. Saccharin/sucralose also markedly decreased microbiota-derived AHR ligands and colonic AHR expression, which are closely associated with many metabolic syndromes. Metformin or fructo-oligosaccharide supplementation significantly restored *A. muciniphila* and AHR ligands in sucralose-consuming mice, consequently ameliorating NAFLD.

**IMPORTANCE** Our findings indicate that the gut-liver signaling axis contributes to saccharin/sucralose consumption-induced NAFLD. Supplementation with metformin or fructo-oligosaccharide is a potential therapeutic strategy for NAFLD treatment. In addition, we also developed a new nutritional strategy by using a natural sweetener (neohesperidin dihydrochalcone [NHDC]) as a substitute for NAS and free sugars.

## INTRODUCTION

Noncaloric artificial sweeteners (NAS) are extensively introduced into commonly consumed drinks and foods worldwide due to their high sweetness, low caloric content, and reduced cost (1). Saccharin and sucralose have been shown to directly pass through the human gastrointestinal tract without undergoing absorption and metabolism (2, 3). They were therefore generally considered to be safe and even exhibit health benefits for weight reduction and normalization of blood sugar levels (4). However, increasing evidence has shown that saccharin, sucralose, and aspartame consumption increase the risk of glucose intolerance and type 2 diabetes in mice and humans by altering gut microbiota composition (5). Long-term consumption of saccharin and sucralose also significantly induced hepatic chronic inflammation, ultimately leading to liver disease (6–8). Although these data are controversial, the U.S. Food and Drug Administration (FDA) approved six NAS products for use. The accepted daily intake (ADI) of saccharin and sucralose in humans is 5 mg/kg of body weight/day in the United States (1). Dietary intake of NAS directly encounters the gut microbiota, which plays key roles in modulating multiple pathophysiological processes (5–8). In-depth exploration of the effects of NAS consumption on human health through cross talk between the host and microbiota is important.

The gut-liver signaling axis contributes to liver diseases, such as alcoholic liver disease (ALD), nonalcoholic fatty liver disease (NAFLD), and hepatocellular carcinoma (HCC) (9, 10). For example, a previous study compared microbiotas from 123 patients with liver cirrhosis and 114 healthy counterparts of Han Chinese origin and revealed 15 biomarkers at the gene and function levels as potential patient discrimination indices using a liver cirrhosis gene catalogue coupled with quantitative metagenomics (11). Another study showed that ethanol exposure depleted intestinal Akkermansia muciniphila abundance in both mice and humans and that A. muciniphila administration successfully improved experimental ALD (12). *A. muciniphila* is a mucin-degrading bacterium residing in the mucus layer and contributes to intestinal barrier integrity and its functions (13). Numerous studies demonstrated that *A. muciniphila* abundance inversely correlates with body weight in mice and humans (14, 15), and its abundance decreases in obese and diabetic mice (16, 17). In addition, direct or indirect *A. muciniphila* administration promoted intestinal barrier function and ameliorated inflammation in mice fed a high-fat diet (HFD) by reducing the systemic endotoxin concentration (17).

NAFLD is generally characterized by hepatic histopathological abnormalities ranging from hepatic steatosis with fat accumulation, nonalcoholic steatohepatitis (NASH), and cirrhosis to HCC and is highly dependent on environmental factors, especially diet (9, 10). Numerous studies have shown that the intestinal microbiome contributes to all stages of NAFLD accompanied by extensive chronic inflammation (10, 18). Saccharin and sucralose consumption may enrich the biosynthesis pathway of microbial products such as lipopolysaccharide (LPS), triggering systemic inflammation and liver diseases (6–8). In diet-induced metabolic syndromes, the microbiota-derived metabolites from dietary sources are important signals and regulators of the host-microbial cross talk; examples are indole metabolites from tryptophan metabolism, which can be potent aryl hydrocarbon receptor (AHR) ligands (19, 20). Reduced production of AHR ligands by the gut microbiota is a key factor in the pathogenesis of metabolic syndromes such as inflammatory bowel disease, obesity, diabetes, and high blood pressure (21, 22). Recent studies also demonstrated that epithelial indoleamine 2,3-dioxygenase 1 (IDO1), one of two rate-limiting enzymes in the tryptophan catabolic pathway, promotes secretory cell differentiation and enhances the mucus layer and mucus-associated microbiota (i.e., *A. muciniphila*) by modulating AHR signaling (23).

Although NAS consumption appears to enhance the risks of many diseases, such as diabetes and liver diseases, the underlying molecular mechanism of NAFLD induced by saccharin/sucralose consumption remains largely unknown. As a natural sweetener, neohesperidin dihydrochalcone (NHDC) is metabolized to innocuous products, and it is classified as a recognized nontoxic compound by the European Union (EU) and the European Food Safety Authority (EFSA) (24–26). NHDC may be a potential alternative due to its high sweetness, lack of caloric content, reduced bitterness, and anti-inflammatory activity (27, 28). Here, we aimed to evaluate the potential impacts of both NAS (saccharin and sucralose) and NHDC consumption on the intestinal microbiota, especially *A. muciniphila* abundance, and on host AHR-associated pathophysiology. These findings provide a potential preventive and therapeutic strategy for human NALFD through recovery of gut *A. muciniphila* and AHR ligands.

## RESULTS

### NAS induce systemic inflammation and NAFLD.

**FIG 1:**
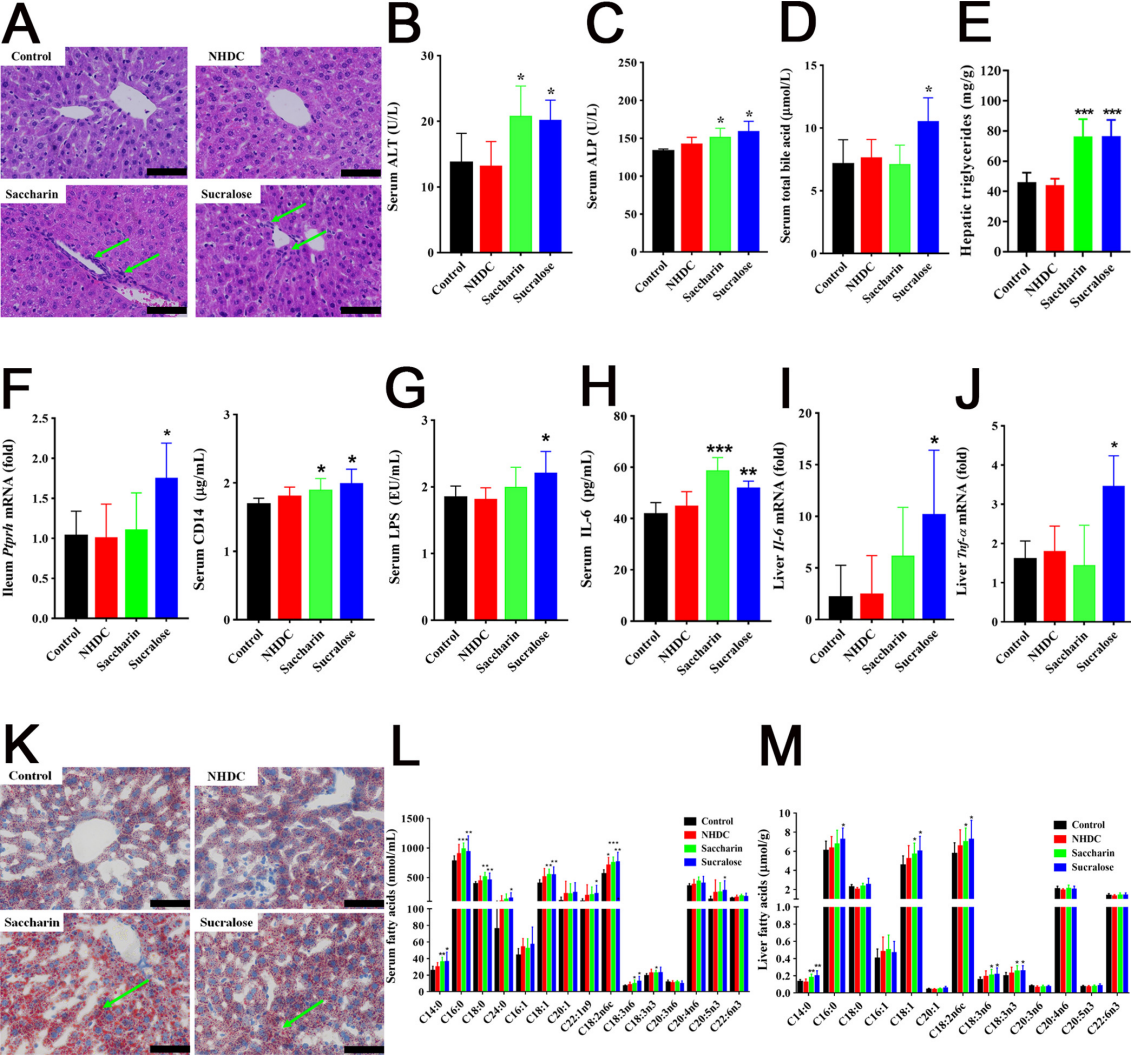
Systemic inflammation and NAFLD induced by saccharin and sucralose consumption. (A) Microscopic images (×400) of H&E-stained liver sections from the control, NHDC, saccharin, and sucralose treatment groups. Arrows indicate inflammatory cell invasion. (B to D) Serum clinical biochemistry analysis of ALT, ALP, and total bile acid. (E) Hepatic triglyceride levels. (F) Gut permeability markers, including soluble CD14 in serum and the level of *Ptprh* mRNA in the ileum. (G) Concentrations of LPS in serum. (H) Concentrations of IL-6 in serum. (I and J) Hepatic levels of *Il-6* and *Tnf-α* mRNA in mice. (K) Histopathological assessment of oil-red-O-stained liver sections. Arrows indicate fat droplets. (L and M) Quantification of fatty acid compositions in the livers and sera of mice. Data are means ± SD (*n* = 10 per group). *, *P < *0.05; **, *P < *0.01; ***, *P < *0.001. Scale bars in panels A and K, 50 μm.

Dietary intake of saccharin and sucralose (0.1 mg/ml) for 11 weeks induced significant systemic inflammation and hepatic lipid accumulation, seen with hematoxylin and eosin (H&E) staining (Fig. 1A) and oil-red-O staining (Fig. 1K), whereas no significant histopathological changes were observed in the livers of mice after NHDC consumption (Fig. 1A and K). Compared with controls, mice with NHDC, saccharin, and sucralose consumption exhibited no marked changes in their volumes of drinking water, food intake, or body weights (see Fig. S1 in the supplemental material). Saccharin and sucralose consumption caused significant elevation of serum alanine transaminase (ALT) and alkaline phosphatase (ALP) levels, indicators of overall liver function (Fig. 1B and C and Table S1). Serum triglyceride (TG) and total bile acid (TBA) levels were significantly increased in the sucralose groups (Fig. 1D and Table S1). Saccharin and sucralose consumption induced significant elevation in the hepatic triglyceride levels of mice (Fig. 1E). Dietary intake of saccharin and sucralose markedly increased mRNA levels of the protein tyrosine phosphatase receptor type H gene (*Ptprh*) in ileum and serum CD14 (Fig. 1F), which are markers of gut permeability closely associated with intestinal barrier function (22, 29). Moreover, NAS and especially sucralose consumption for 11 weeks caused a significant elevation in the levels of serum LPS, serum interleukin 6 (IL-6), and hepatic proinflammatory cytokines, such as *Tnf-α* and *Il-6* (Fig. 1G to J). Targeted compositional measurements of long-chain fatty acids (LCFAs) showed that saccharin and sucralose markedly elevated levels of saturated fatty acids (SFA; C14:0, C16:0, C18:0, and C24:0), monounsaturated fatty acids (MUFA; C18:1 and C22:1n9), and polyunsaturated fatty acids (PUFA; C18:2n6c, C18:3n6, C18:3n3, C20:5n3) in the sera of mice (Fig. 1L). Significantly elevated SFA (C14:0 and C16:0), MUFA (C18:1), and PUFA (C18:2n6c, C18:3n6 and C18:3n3) were also observed in the livers of mice after saccharin and sucralose consumption for 11 weeks (Fig. 1M). Furthermore, untargeted ^1^H nuclear magnetic resonance (NMR)-based metabolomics showed that saccharin and sucralose induced lipid accumulation in the liver (Fig. S2). It is noteworthy that NHDC consumption had minimal effects, including no change in LPS level, inflammatory cytokines, and lipid metabolism in the livers or sera of mice (Fig. 1).

10.1128/mSystems.00985-20.2 FIG S1 Physiological parameters characterized in mice from the control, NHDC, saccharin, and sucralose treatment groups. (A) Liquid intake. (B) Chow consumption. (C) Body weight. Data are means ± SD (*n* = 10 per group). *, *P < *0.05; **, *P < *0.01; ***, *P < *0.001. Download FIG S1, TIF file, 0.4 MB. Copyright © 2021 Shi et al. 2021 Shi et al. https://creativecommons.org/licenses/by/4.0/ This content is distributed under the terms of the Creative Commons Attribution 4.0 International license.

10.1128/mSystems.00985-20.3 FIG S2 Orthogonal partial least-squares discriminant analysis (OPLS-DA) scores (left) and coefficient-coded loading plots for the models (right) from NMR spectra of livers. OPLS-DA plots discriminate between the control (green dots) and NHDC (blue dots), saccharin (red dots), or sucralose (yellow dots) groups. These models are cross-validated with coefficient of variation (CV) ANOVA (*P = *0.08, *P = *0.0019, and *P = *0.000016) for the models of mouse liver after NHDC, saccharin, and sucralose treatment, respectively. Download FIG S2, TIF file, 0.3 MB. Copyright © 2021 Shi et al. 2021 Shi et al. https://creativecommons.org/licenses/by/4.0/ This content is distributed under the terms of the Creative Commons Attribution 4.0 International license.

10.1128/mSystems.00985-20.9 TABLE S1 Clinical biochemistry parameters in the sera of mice after NHDC, saccharin, or sucralose consumption. Download Table S1, DOCX file, 0.02 MB. Copyright © 2021 Shi et al. 2021 Shi et al. https://creativecommons.org/licenses/by/4.0/ This content is distributed under the terms of the Creative Commons Attribution 4.0 International license.

### NAS disrupt the gut microbiota with depletion of *A. muciniphila* abundance.

**FIG 2:**
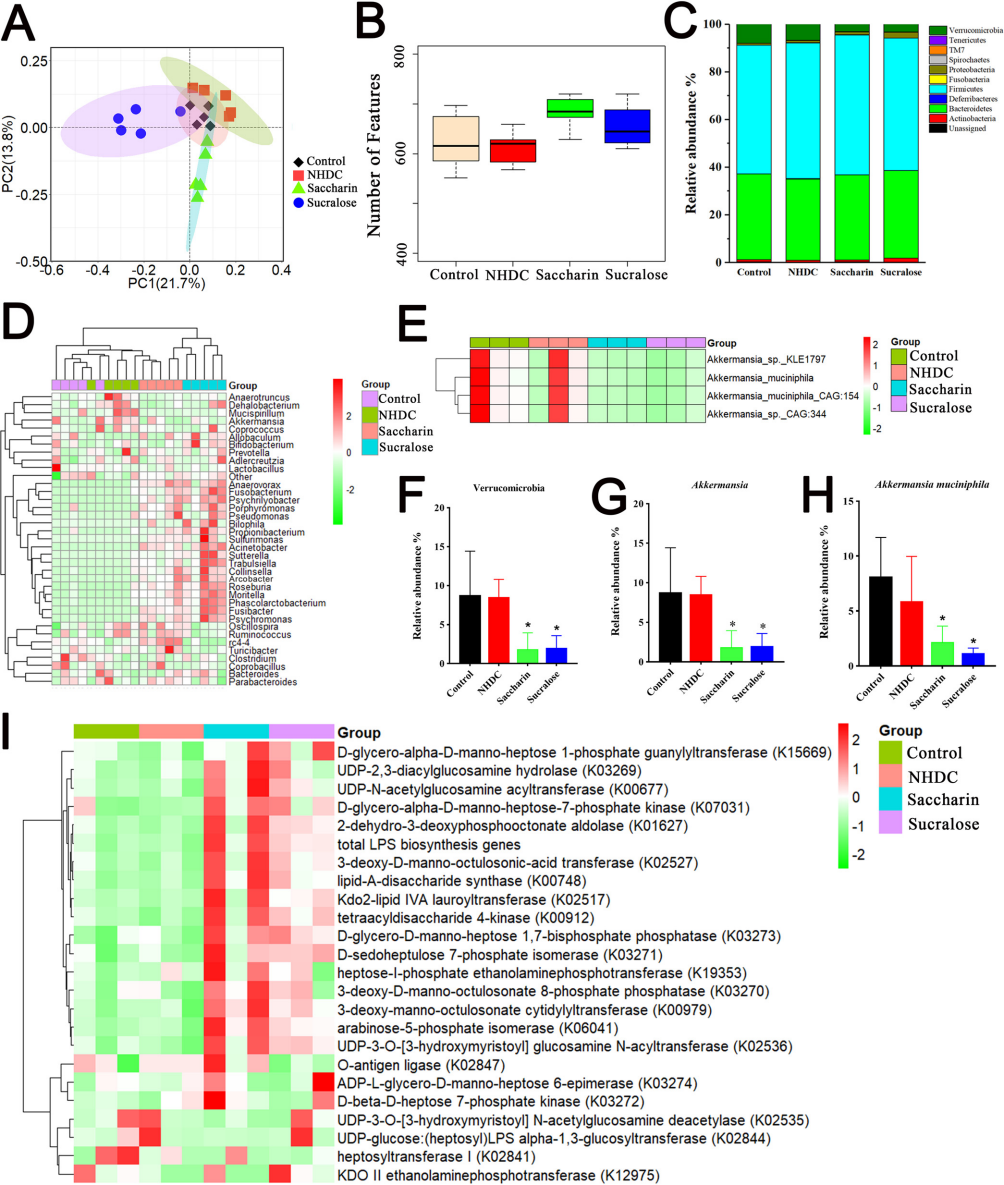
Altered gut microbial community structure with depletion of *A. muciniphila* abundance in saccharin- and sucralose-treated mice. (A) β-Diversity index analysis based on Bray-Curtis dissimilarities. PC1 and PC2, principal components 1 and 2, respectively. (B) α-Diversity analysis based on numbers of features. (C) Relative abundances of gut microbes at the phylum level. (D) Heatmap of taxon abundances at the genus level. (E) Metagenomic shotgun sequencing analysis of *Akkermansia* at the species level. (F) 16S rRNA gene sequencing analysis of *Verrucomicrobia* abundance. (G) 16S rRNA gene analysis of the abundance of the genus *Akkermansia*. (H) Metagenomic analysis of *A. muciniphila* abundance. (I) Metagenomic functional analysis of LPS biosynthetic genes. Data are means ± SD. *, *P < *0.05; **, *P < *0.01; ***, *P < *0.001.

Given that NAS consumption directly encounters the gut microbial community (2, 3), we aimed to determine the effects of NAS on the composition of the gut microbiota and identify specific bacterial strains/species relating to hepatic inflammation and steatosis. The principal-coordinate analysis (PCoA) based on Bray-Curtis distances indicated that saccharin (*R^2^* = 0.584, *P = *0.00894) and sucralose (*R^2^* = 0.62, *P = *0.00891) induced a significant change in the community structure of the gut microbiota in the cecal contents of mice, whereas NHDC had no significant impact on the bacterial community (*R^2^* = 0.2, *P = *0.1) (Fig. 2A). Microbial α-diversity analysis showed that the unique α-diversity indices between the control, NHDC, and saccharin or sucralose groups were not significantly different (Fig. 2B). Within the intestinal microbiota, *Firmicutes*, *Bacteroidetes*, *Actinobacteria*, *Proteobacteria*, and *Verrucomicrobia* were observed as the dominant bacteria at the phylum level (Fig. 2C). Saccharin and sucralose consumption profoundly increased the levels of *Proteobacteria* and *Actinobacteria* in the cecal contents of mice (Fig. S3A and B). At the genus level, comparisons between bacterial community characteristics were tabulated and clustered using a heatmap representing the relative abundance of each genus (Fig. 2D). The results showed that NAS groups (especially the sucralose group) were clearly separated from controls, suggesting the different community characteristics between them. However, NHDC and control groups were clustered together as showing their similar microbial community characteristics (Fig. 2D). Of particular note, the abundances of the genus *Akkermansia* (Fig. 2G) and all higher taxonomies, such as the phylum *Verrucomicrobia* (Fig. 2F), were significantly downregulated in the groups receiving saccharin and sucralose in comparison with those of the control and NHDC groups. Significantly elevated abundances of the genera *Streptococcus*, *Prevotella*, *Fusibacter*, *Lachnospira*, *Anaerovorax*, *Psychrilyobacter*, *Psychromonas*, and *Trabulsiella* and the depleted genus *Lactobacillus* were observed in the cecal contents of mice after saccharin or sucralose consumption (Fig. S3C to L). It is worth noting that saccharin and sucralose consumption significantly reduced or increased the abundances of numerous taxonomic groups, as revealed by cladogram analysis using LEfSe (Fig. S4).

10.1128/mSystems.00985-20.4 FIG S3 Gut microbiota dysbiosis in NAS-consuming mice. (A and B) Significantly changed taxa at the phylum level. (C to L) Significantly changed taxa at the genus level. Data are means ± SD (*n* = 6 per group). *, *P < *0.05; **, *P < *0.01; ***, *P < *0.001. Download FIG S3, TIF file, 0.4 MB. Copyright © 2021 Shi et al. 2021 Shi et al. https://creativecommons.org/licenses/by/4.0/ This content is distributed under the terms of the Creative Commons Attribution 4.0 International license.

10.1128/mSystems.00985-20.5 FIG S4 Analysis of the relative abundances of taxa using LEfSe indicates multiple taxa, including the genus *Akkermansia*. The taxonomic groups displayed at the right are differentially enriched in the groups: control (red), NHDC (green), saccharin (blue), and sucralose (purple). Download FIG S4, TIF file, 2.8 MB. Copyright © 2021 Shi et al. 2021 Shi et al. https://creativecommons.org/licenses/by/4.0/ This content is distributed under the terms of the Creative Commons Attribution 4.0 International license.

Metagenomic shotgun sequencing further showed that saccharin and sucralose consumption induced marked depletion in the abundance of *A. muciniphila*, which is the most dominant species in the genus *Akkermansia* (>98%) (Fig. 2E and H). Metagenomic functional analysis of bacterial proinflammatory genes showed that LPS biosynthetic genes were significantly elevated in saccharin- or sucralose-treated mice (Fig. 2I and Fig. S5). In addition, significantly decreased levels of short-chain fatty acids (SCFAs), such as propionic acid and butyric acid, were observed in the cecal contents of mice after respective saccharin and sucralose consumption, whereas NHDC consumption significantly increased levels of propionic acid and isobutyric acid (data not shown).

10.1128/mSystems.00985-20.6 FIG S5 KEGG pathways and functional structures of the metagenome. (A) Histogram of KEGG pathways. (B) Functional classification according to the cluster of orthologous group (COG) categories. Download FIG S5, TIF file, 1.1 MB. Copyright © 2021 Shi et al. 2021 Shi et al. https://creativecommons.org/licenses/by/4.0/ This content is distributed under the terms of the Creative Commons Attribution 4.0 International license.

### NAS reduce microbiota-derived AHR ligands.

**FIG 3:**
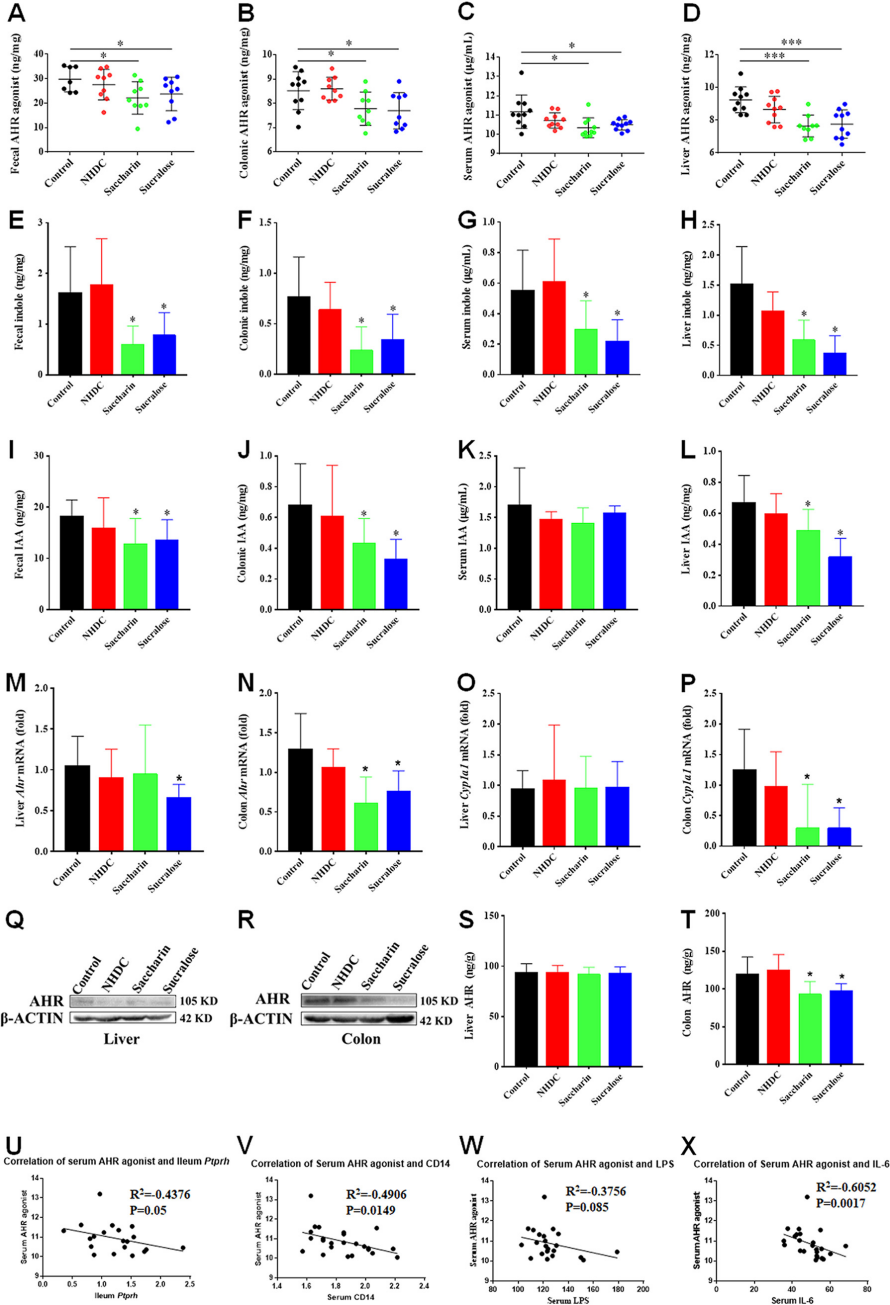
Impaired microbiota-derived AHR ligands in saccharin- and sucralose-treated mice. (A to D) Total concentrations of AHR agonists (indole, IAA, IPA, indole acrylic acid, and indole-3-aldehyde) from fecal, colonic, serum, and liver samples of the control, NHDC, saccharin, and sucralose treatment groups. (E to H) Concentrations of indole from fecal, colonic, serum, and liver samples. (I to L) Concentrations of IAA from fecal, colonic, serum, and liver samples. (M and N) Hepatic and colonic levels of *Ahr* mRNA. (O and P) Hepatic and colonic levels of *Cyp1a1* mRNA. (Q and R) Levels of hepatic and colonic AHR protein. (S and T) Hepatic and colonic AHR activity. (U to X) Spearman correlations of serum AHR agonists, ileum *Ptprh*, and serum CD14, LPS, and IL-6. Data are means ± SD (*n* = 10 per group). *, *P < *0.05; **, *P < *0.01; ***, *P < *0.001.

Targeted analysis of tryptophan metabolites showed that saccharin and sucralose consumption induced marked depletion in the levels of AHR agonists (Fig. 3A to D), manifested by significantly decreased levels of indole (Fig. 3E to H), indole acetic acid (IAA) (Fig. 3I to L), and indole-3-propionic acid (IPA) (Fig. S6A to D) in the feces, colon, serum, and liver specimens of mice. Indole, IAA, IPA, indole acrylic acid, and indole-3-aldehyde are commonly regarded as microbiota-derived AHR ligands (30). Consistently, sucralose consumption significantly decreased levels of AHR mRNA in the livers and proximal colons of mice (Fig. 3M and N). However, significant depletion in the level of the AHR protein was not observed in the livers but was observed in the colons of mice following saccharin and sucralose consumption (Fig. 3Q and R). NAS selectively downregulated colonic AHR activity (Fig. 3S and T) and its target gene *Cyp1a1* (Fig. 3O and P). Furthermore, correlation analysis showed that serum AHR agonists negatively correlated with gut permeability markers (serum CD14, *P = *0.0149; ileal *Ptprh*, *P = *0.05) and inflammatory factors (serum IL-6, *P = *0.0017; serum LPS, *P = *0.085) (Fig. 3U to X). In addition, kynurenine (KYN), one of the tryptophan metabolites and a key contributor to the development of chronic inflammatory diseases (31, 32), was significantly increased in fecal samples of the sucralose group (Fig. S6E to H).

10.1128/mSystems.00985-20.7 FIG S6 Quantification of tryptophan metabolites based on UHPLC-QQQ MS. (A to D) Concentrations of tryptophan metabolites through indole/AHR pathways (indole, IAA, IA, IPA, IPV, ILA, IAID, and tryptophol) from fecal, colonic, serum, and liver samples of the control, NHDC, saccharin, and sucralose treatment groups. (E to H) Concentrations of tryptophan metabolites through kynurenine/IDO pathways (Trp, Quin, 3HKN, XAN, 3HAA, KYN, and KA) from fecal, colonic, serum, and liver samples. Data are means ± SD (*n* = 10 per group). *, *P < *0.05; **, *P < *0.01; ***, *P < *0.001. IAA, indole acetic acid; IPA, indole-3-propionic acid; IA, indole acrylic acid; IAID, indole-3-aldehyde; IPV, indole-3-pyruvate; ILA, indole-3-lactic acid; Trp, tryptophan; Quin, quinolinic acid; 3HKN, 3-hydroxykynurenine; XAN, xanthurenic acid; 3HAA, 3-hydroxyanthranilic acid; KYN, kynurenine; KA, kynurenic acid. Download FIG S6, TIF file, 0.4 MB. Copyright © 2021 Shi et al. 2021 Shi et al. https://creativecommons.org/licenses/by/4.0/ This content is distributed under the terms of the Creative Commons Attribution 4.0 International license.

**FIG 4:**
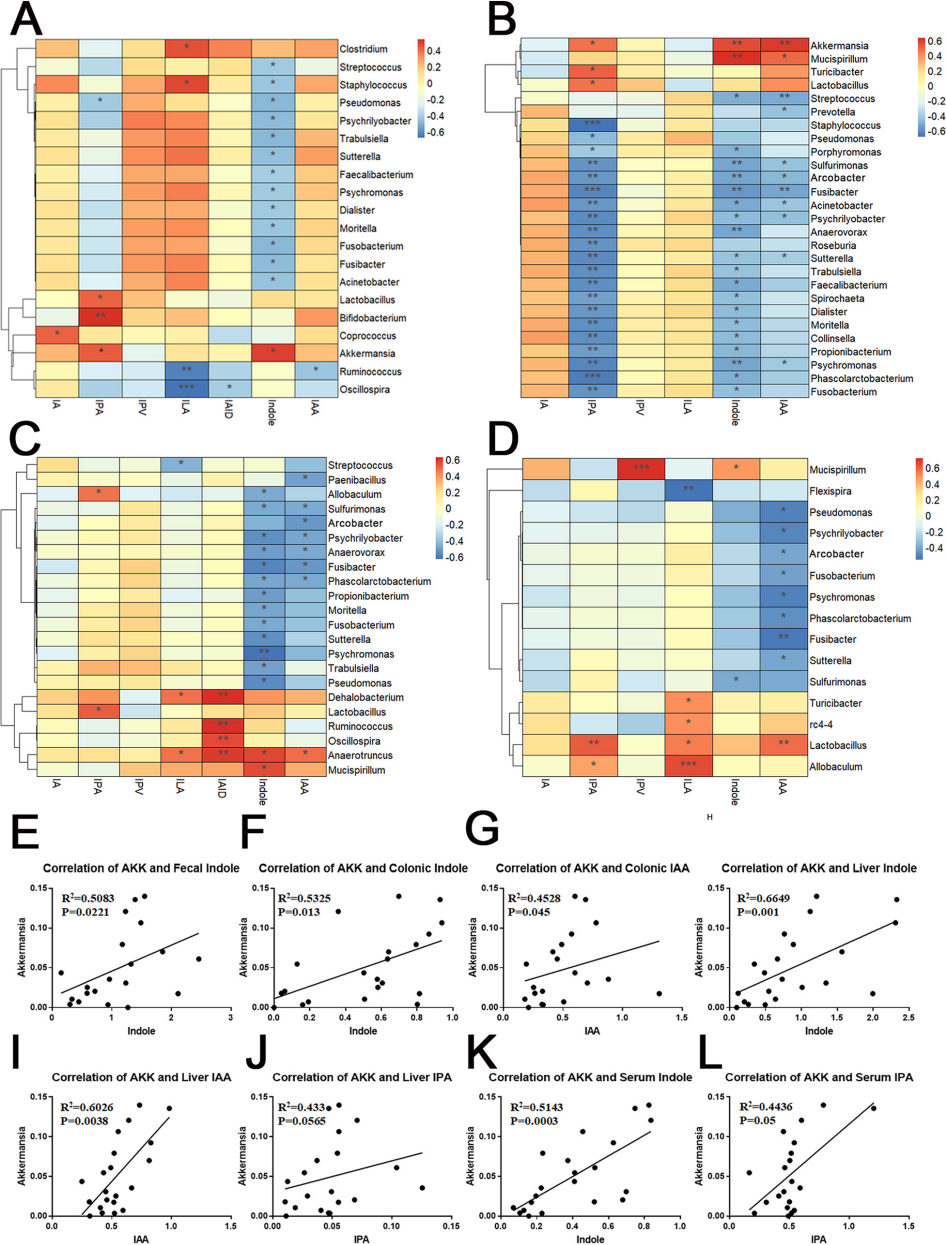
Correlation analysis between the gut microbiota and microbiota-derived indole metabolites from tryptophan metabolism. (A to D) Correlation heatmaps based on Spearman correlation analysis from serum, liver, fecal, and colonic samples. (E to L) Correlation analysis between the abundance of the genus *Akkermansia* and the microbiota-derived AHR ligands. AKK, *Akkermansia*. Data are means ± SD (*n* = 6 per group). *, *P < *0.05; **, *P < *0.01; ***, *P < *0.001.

To explore the relationship between the gut microbiota (47 genera) and microbiota-derived AHR ligands, correlation heatmaps were obtained by calculating Spearman’s rank correlation coefficient of all genera and indole metabolites from tryptophan in serum, liver, fecal, and colonic samples (Fig. 4A to D). Of particular note, the genus *Akkermansia* was significantly positively correlated with microbiota-derived AHR ligands in all four tissues (Fig. 4E to L), further suggesting that depletion of *A. muciniphila* abundance is closely associated with the impaired microbiota-derived AHR ligands in saccharin- and sucralose-treated mice. Simultaneously, the results also revealed that the abundance of the genus *Akkermansia* was significantly negatively correlated with the levels of gut permeability markers, LPS, inflammatory factors, and LCFAs in serum and liver and significantly positively correlated with SCFAs (propionic acid and butyric acid) (Fig. S7A to O).

10.1128/mSystems.00985-20.8 FIG S7 Spearman correlation of the abundance of the genus *Akkermansia* and the levels of ileum *Ptprh*, serum Cd14 and LPS, hepatic inflammatory factors, SCFAs in cecal contents, and LCFAs in serum and liver. AKK, *Akkermansia*. Download FIG S7, TIF file, 0.3 MB. Copyright © 2021 Shi et al. 2021 Shi et al. https://creativecommons.org/licenses/by/4.0/ This content is distributed under the terms of the Creative Commons Attribution 4.0 International license.

### Supplementation with metformin or FOS restores *Akkermansia* and AHR ligands.

**FIG 5:**
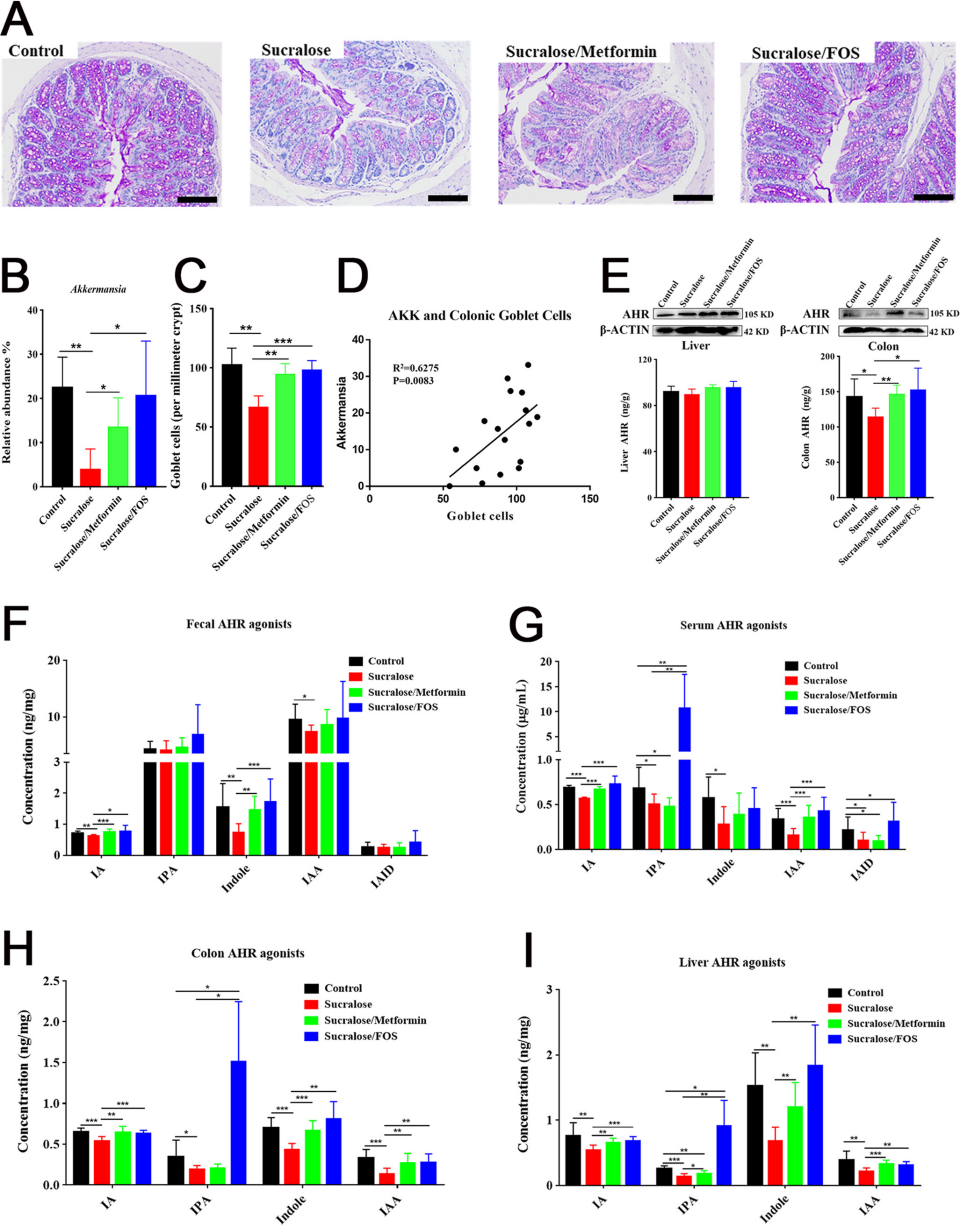
Supplementation with metformin or FOS restores *Akkermansia* abundance and AHR ligands and increases the number of goblet cells of sucralose-treated mice. (A) Representative periodic acid-Schiff (PAS) stains of colons. Scale bars, 100 μm. (B) Genus *Akkermansia* abundance in control, sucralose, sucralose/metformin, and sucralose/FOS treatment groups. (C) Numbers of colonic goblet cells. (D) Spearman correlation of *Akkermansia* (AKK) abundance and the number of colonic goblet cells. (E) Hepatic and colonic AHR protein levels and AHR activity. (F to I) Concentrations of AHR ligands from fecal, serum, colonic, and liver samples, respectively. Data are means ± SD (*n* = 10 per group). *, *P < *0.05; **, *P < *0.01; ***, *P < *0.001.

Dietary metformin or fructo-oligosaccharide (FOS) as normally used for supplementation of *Akkermansia* was further employed to ameliorate NAFLD induced by sucralose consumption (17, 33). The results showed that supplementation with metformin or FOS significantly restored *Akkermansia* abundance and subsequently enhanced the number of goblet cells in the colons of sucralose-treated mice (Fig. 5A to C). We also found that a strongly positive correlation occurred between *Akkermansia* abundance and the number of colonic goblet cells based on Spearman correlation analysis (Fig. 5D). Supplementation with metformin or FOS also specifically restored the downregulated AHR activity and protein levels of AHR in the colons of mice induced by sucralose consumption (Fig. 5E), which were further confirmed with the restored levels of tryptophan-based AHR ligands, such as indole metabolites by metformin or FOS supplementation (Fig. 5F to I).

### Supplementation with metformin or FOS improves NAFLD.

**FIG 6:**
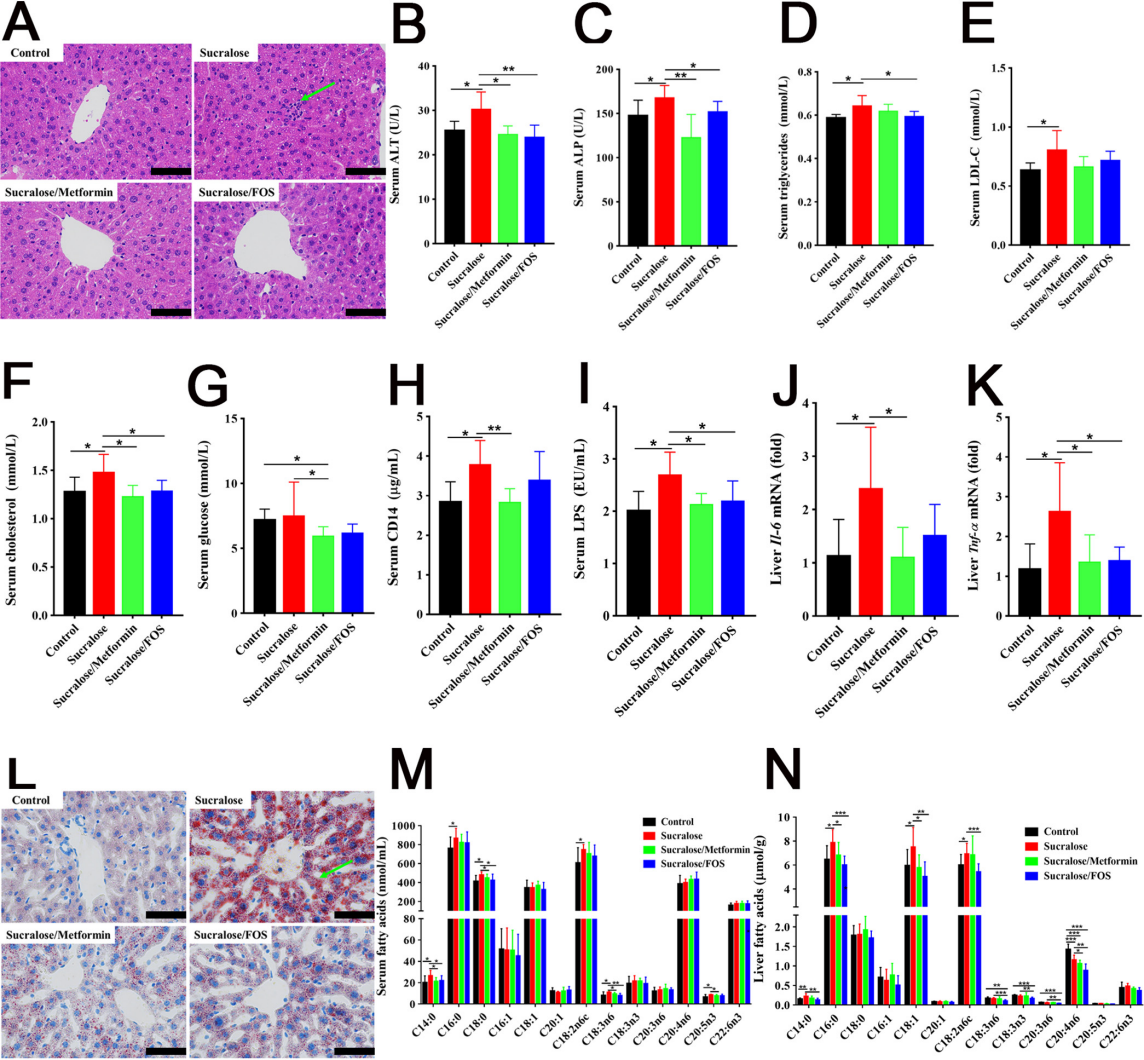
Supplementation with metformin or FOS improves systemic inflammation and NAFLD by sucralose exposure. (A) Microscopic images (×400) of H&E-stained liver sections from control, sucralose, sucralose/metformin, and sucralose/FOS treatment groups. Arrows indicate inflammatory cell invasion. (B to G) Clinical biochemistry analyses of serum ALT, ALP, triglyceride, LDL, cholesterol, and glucose. (H and I) Concentrations of serum CD14 and LPS. (J and K) Hepatic levels of *Il-6* and *Tnf-α* mRNA of mice. (L) Histopathological assessment of oil-red-O-stained liver sections. Arrows indicate fat droplets. (M and N) Quantifications of fatty acid compositions in the sera and livers of mice, respectively. Data are means ± SD (*n* = 10 per group). *, *P < *0.05; **, *P < *0.01; ***, *P < *0.001. Scale bars in panels A and L, 50 μm.

Given our recovery of *Akkermansia* organisms, the colonic AHR level, and the number of goblet cells in the metformin or FOS intervention mice, we next assessed hepatic inflammation and steatosis in sucralose-treated mice. Sucralose-treated mice supplemented with metformin or FOS exhibited significant improvements in the levels of liver function indicators, including serum ALT, ALP, TG, low-density lipoproteins (LDL), cholesterol, and glucose (Fig. 6B to G and Table S2). The systemic inflammation of sucralose-consuming mice was also ameliorated by supplementation of metformin or FOS, manifested by significant improvements in the histopathological changes and levels of serum CD14, LPS, and hepatic proinflammatory cytokines (e.g., *Tnf-α* and *Il-6*) (Fig. 6A and H to K). The oil-red-O staining showed that the lipid accumulation in sucralose-consuming mice was greatly reduced by supplementation with metformin or FOS (Fig. 6L), indicating that NAFLD induced by sucralose consumption was highly improved. This observation was further verified with observations of significant reductions in the levels of SFA (C14:0 and C18:0) and PUFA (C18:3n6 and C20:5n3) in the sera of sucralose-treated mice (Fig. 6M). Marked restoration of the levels of SFA (C14:0 and C16:0), MUFA (C18:1), and PUFA (C18:2n6c) was also observed in the livers of sucralose-treated mice after supplementation with metformin or FOS (Fig. 6N). Collectively, restoration of the levels of serum CD14 and LPS, proinflammatory cytokines, SCFAs, and LCFAs contributes to the improved inflammation and steatosis in the livers of sucralose-treated mice after metformin or FOS supplementation.

10.1128/mSystems.00985-20.10 TABLE S2 Clinical biochemistry parameters in the sera of mice after sucralose, sucralose/metformin, or sucralose/FOS treatments. Download Table S2, DOCX file, 0.02 MB. Copyright © 2021 Shi et al. 2021 Shi et al. https://creativecommons.org/licenses/by/4.0/ This content is distributed under the terms of the Creative Commons Attribution 4.0 International license.

## DISCUSSION

Most noncaloric artificial sweeteners (NAS) pass through the human gastrointestinal tract without absorption or metabolism and directly encounter the gut microbiota community (2, 3). Increasing evidence has shown that cross talk between the host and the gut microbiota plays a pivotal role in the development of prevalent metabolic diseases, such as NAFLD, diabetes, and cancer (9–12). NAS consumption has been discovered to induce many adverse metabolic effects (e.g., glucose intolerance) in mice and humans by disrupting the gut microbiota (5–8), thus arousing considerable public concern. Here, we provide a hypothesis. Does diet change the gut microbiome, which in turn skews the immune system via lack of AHR activation and then promote inflammation and disease? Using a highly innovative combination of metagenomics, metabolomics, and biological assays, we here reveal that NAS (saccharin and sucralose) consumption causes pronounced gut microbiota compositional changes and systemic inflammation-triggered NAFLD through the gut-liver signaling axis. Notably, significant depletion of the abundance of the commensal *A. muciniphila* (phylum *Verrucomicrobia*) was observed in the cecal contents of mice following saccharin and sucralose consumption. *A. muciniphila*, an intestinal mucin-degrading bacterium, is highly associated with the mucus layer by using mucin as its sole nutritional source of carbon and nitrogen (13). Numerous studies have demonstrated that *A. muciniphila* supports the maintenance of the intestinal barrier integrity and exhibits probiotic properties with anti-inflammatory effects and even promotes the efficacy of adjuvant immunotherapy of cancer (16, 17, 34). However, *A. muciniphila* can also have negative effects in certain scenarios. Multiple studies have implicated *A. muciniphila* in infectious disease, where *A. muciniphila* enhances the pathogenicity of mucosal pathogens, including Citrobacter rodentium (35), Clostridium difficile (36), and Salmonella enterica (37). Additionally, Everard et al. demonstrated that *A. muciniphila* increased mucus in mice on a high-fat diet but that *A. muciniphila* slightly reduced the inner mucus layer of specific-pathogen-free (SPF) mice on a normal chow (17).

In the current study, significant depletion of *A. muciniphila* abundance by NAS consumption expectedly reduced the number of mucin-producing goblet cells, accompanied by disruption of intestinal barrier integrity. Consequently, the gut permeability of mice was altered by NAS consumption, leading to gut leakiness. Microbial products, such as bacterial endotoxin LPS, cross the mucosa and subsequently spread to the whole body through the portal vein and blood circulation. These results were highly consistent with previous observations of ALD development (12). Significant reductions in *A. muciniphila* abundance have also been discovered in both fecal samples and mucosal biopsy specimens of patients with ulcerative colitis (38). Here, markedly elevated levels of serum LPS and bacterial LPS biosynthetic genes in the cecal contents of mice suggested that NAS triggered host immune responses with high levels of systemic inflammatory signaling, leading to NAFLD. Supportive evidence of NAFLD by saccharin and sucralose through the gut-liver signaling axis could also be found in significant TG and fat accumulation in the livers or sera of mice.

AHR activated by exogenous or endogenous ligands is involved in xenobiotic and immune responses and the pathogenesis of metabolic diseases, such as obesity and cancer (39–41). AHR genetic (*AhR*^−/−^ and *AhR^−/+^*) mice have been employed to comparatively investigate the possible roles of AHR in various physiological and pathological processes, including diet-induced metabolic diseases (42). Independently of the gut microbiota, some studies showed that exogenous ligand (e.g., dioxin) exposure induced toxicity, teratogenicity, and carcinogenicity by activating AHR (29, 43). However, metabolic syndromes, such as obesity, diabetes, and high blood pressure of humans and animal models exhibit reduced AHR agonist activity, as shown by lower gut microbiota-derived AHR agonist production, such as of tryptophan metabolites (22). In this study, NAS significantly downregulated the levels of AHR agonists in fecal, colonic, serum, and liver samples of mice. Concomitantly, colonic AHR expression at both the mRNA and the protein level was selectively reduced, whereas no significant changes in AHR protein level were observed in the livers of mice after NAS consumption. One possible explanation of selective reduction in the protein levels of colonic AHR may be that these AHR ligands are derived from tryptophan metabolism by gut microbiota. Specifically, gut microorganisms metabolize tryptophan to a variety of indole compounds, most of which are AHR ligands promoting intestinal homeostasis through the activation of AHR.

Here, both *A. muciniphila* and microbiota-derived AHR agonists were negatively correlated with levels of gut permeability markers, serum LPS, and inflammatory cytokines (IL-6), suggesting that there is a close relationship between AHR ligands and *A. muciniphila* abundance involved in the occurrence and development of NAS-induced NAFLD. Actually, recent studies revealed that a high-fat diet (HFD) concurrently decreased AHR metabolic activity and *A. muciniphila* abundance in mice (22). Epithelial indoleamine 2,3-dioxygenase 1 (IDO1) inhibits activation of colonic Notch signaling by interacting with the AHR, thus promoting secretory cell differentiation and increasing thickness of intestinal mucus layers and proportions of *A. muciniphila* abundance, which reduce inflammatory disease severity (23). The AHR has been shown to play a critical role in regulating intestinal homeostasis and protecting the host from the environment through maintenance of intestinal barrier function (39). Therefore, it can be speculated that both *A. muciniphila* abundance and microbiota-dependent AHR signaling may contribute to the pathogenesis of the liver inflammation and steatosis of mice induced by NAS consumption through gut-liver interaction. Genetic AHR and germfree animal models will warrant further investigation to address the challenges to the relationship between AHR ligands, *A. muciniphila*, and associated metabolic diseases.

The AHR is a ligand-activated transcription factor which can be activated by dietary and microbial aromatic molecules. AHR activation by AHR agonists has been shown to play a critical role in promoting repair, inhibiting inflammation, and maintaining intestinal homeostasis in the intestinal mucosa (44, 45). The protective effects of AHR activation in the intestinal tract have been postulated by promoting cytokines such as IL-22 and IL-10 on immune cells. Recent studies showed that microbiota-derived AHR agonists from tryptophan, including indole-3-aldehyde and 6-formylindolo [3,2-b]carbazole, provided intestinal mucosa protection in mice by promoting IL-22, which is the target gene of AHR activation (20, 46). Inefficiency of the gut microbiota to produce microbiota-derived AHR agonists from tryptophan was closely associated with the pathogenesis of inflammatory bowel disease, notably through impaired IL-22 (21). In addition, it was reported that IPA potently prevented chronic inflammation of the colon in mice by upregulating the expression of IL-10 and activating AHR (47).

Metformin, a typical drug used for diabetes and obesity (48, 49), and FOS as an effective prebiotic (50, 51) have normally been used for amelioration of diabetes, obesity, and NAFLD. Here, supplementation with metformin or FOS restored the genus *Akkermansia* abundance, increased the number of mucus-producing goblet cells, and rescued impaired AHR ligands in mice after NAS consumption. Consequently, NAS-induced phenotypes, such as hepatic inflammation and steatosis, were markedly ameliorated, manifested by recovery of the serum LPS level and reductions in the accompanying systemic inflammation and lipid dysmetabolism of mice after supplementation with metformin or FOS. As in our study, metformin had been employed to replenish the abundance of *A. muciniphila* to improve glucose homeostasis in HFD-fed obese mice (33, 52). Administration of FOS increased the abundance of *A. muciniphila* by 100-fold in genetically obese mice (15) and increased the number of goblet cells and the thickness of the mucus layer with abolishment of metabolic endotoxemia in rats (53). Furthermore, direct oral *A. muciniphila* supplementation was previously reported to ameliorate experimental alcoholic liver disease (ALD) by protecting against ethanol-induced gut leakiness and enhancing intestinal barrier integrity (12). These observations indicated that increased *A. muciniphila* abundance has beneficial effects with regard to the maintenance of epithelial barrier integrity and protection from inflammatory intestinal conditions. Thus, direct or indirect *A. muciniphila* supplementation may be an effective treatment strategy for metabolic diseases, such as NAFLD and diabetes.

**FIG 7:**
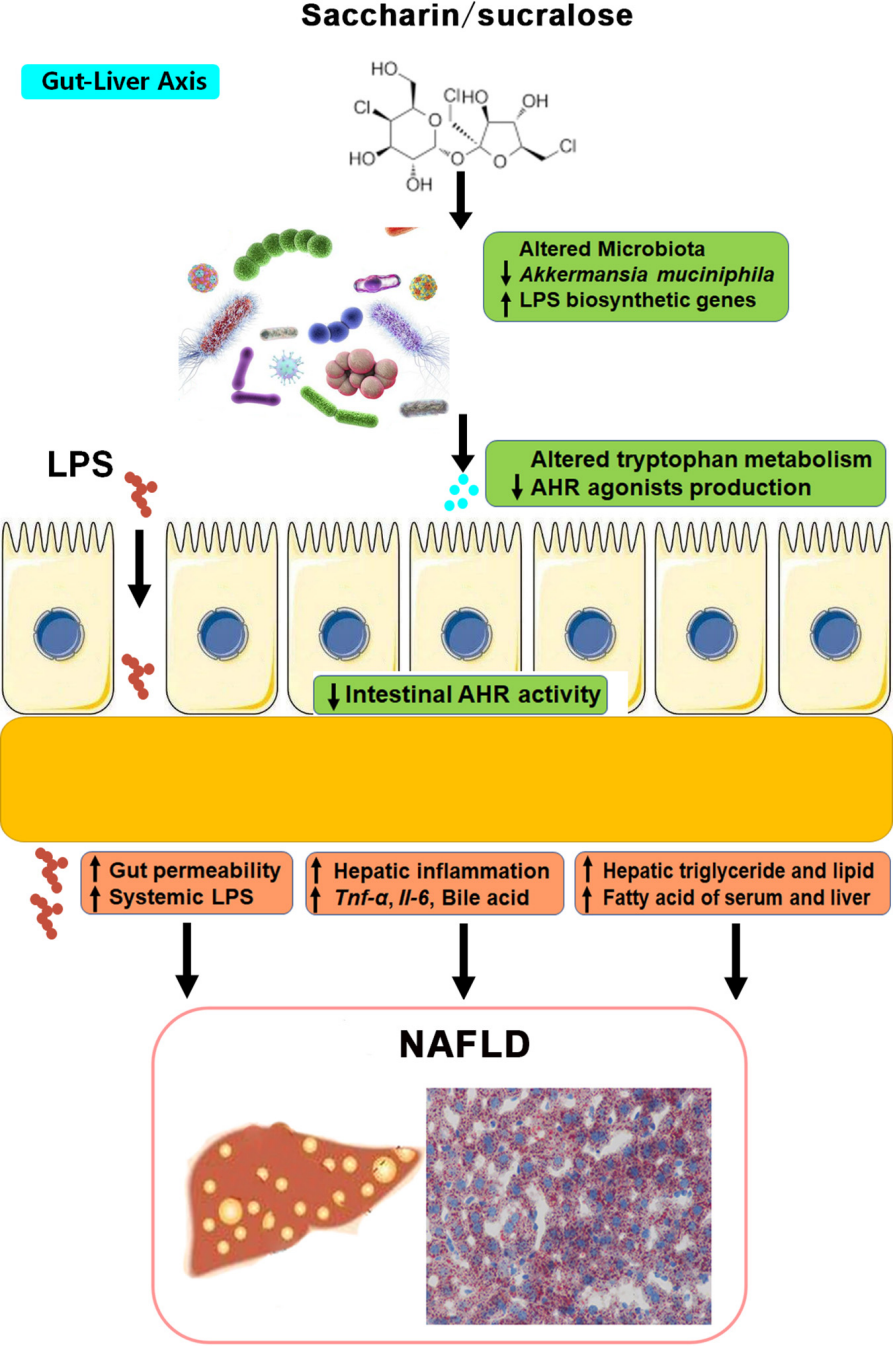
Summary of the molecular mechanisms of saccharin and sucralose consumption-induced NAFLD in mice through gut-liver cross talk. Long-term saccharin/sucralose consumption altered the gut microbial community structure and composition, significantly depleted *A. muciniphila* abundance, and elevated LPS biosynthetic genes in the gut microbiotas of mice, resulting in gut leakiness and a high level of serum LPS, which caused systemic inflammation and NAFLD in mice. Saccharin/sucralose also markedly decreased microbiota-derived AHR ligands and colonic AHR expression, which was closely correlated with metabolic syndromes.

In conclusion, these findings revealed that long-term consumption of saccharin and sucralose has adverse health effects contributing to the occurrence and development of NAFLD through the gut-liver signaling axis (Fig. 7). Supplementation with metformin or FOS may be developed as a potential preventive and therapeutic strategy in patients with NAFLD through recovery of *A. muciniphila* abundance and AHR ligands. A new nutritional strategy with a natural sweetener (NHDC) may be developed as a substitute for NAS and free-sugar consumption.

## MATERIALS AND METHODS

### Animal experiments.

Animal experimental procedures were performed according to the Chinese National Guidelines and approved by the animal ethics committee of the Wuhan Institute of Physics and Mathematics (WIPM; China). A total of 40 female C57BL/6 mice (6 weeks old, 20- to 25-g body weight) were purchased from the Charles River Laboratories (Beijing, China) and allowed to acclimate in the facility for 1 week. All mice were housed in a specific-pathogen-free room with a 12-h light/dark cycle and a constant temperature (22°C ± 1°C) and humidity (40 to 60%) at the Wuhan Institute of Virology (Hubei, China). In the NAS exposure experiment, mice were randomly divided into four groups (*n* = 10), fed a standard normal-chow diet, and given sterilized water or fresh sterilized solution containing NHDC, saccharin, or sucralose. Experiments conducted with a pure NHDC, saccharin, and sucralose solution of 0.1 mg/ml were used to meet the FDA-approved acceptable daily intake (ADI) in humans (5 mg/kg/day) based on NAS exposure doses previously calculated (5). In the studies of supplementation with metformin or FOS, a total of 40 C57BL/6 female mice were also randomly divided into four groups (*n* = 10) that received sterilized water or fresh sterilized solution containing sucralose, sucralose with metformin, or sucralose with FOS. The concentration of sucralose solution was 0.1 mg/ml, and the two supplementation groups of mice were subjected to 0.4 mg/ml of metformin or 50 mg/ml of FOS (54). Animal experiments of NAS exposure and supplementation with metformin or FOS were continued through drinking water consumption for 11 and 10 weeks, respectively. Food intake, water intake, and the body weights of mice were monitored and recorded every week. Urine and fecal samples were collected every week over the experimental period. At the end of the experiments, the mice were sacrificed under isoflurane anesthesia after 8 h of fasting. All the samples, including serum, liver, colon, ileum, and cecal content samples, were immediately collected and stored at −80°C for the following experiments.

### Histopathology and clinical biochemistry.

Following mouse sacrifice, part of the liver tissues was fixed in 10% formalin solution. The fixed liver biopsy specimens were embedded in paraffin wax, sectioned (3 to 4 μm), and stained with H&E and oil-red-O stain. Part of the proximal colon tissues were fixed in 10% formalin solution for periodic acid-Schiff (PAS) staining. Part of the serum samples were used for clinical biochemistry analysis within 24 h, including assessments of alanine aminotransferase (ALT), alkaline phosphatase (ALP), aspartate transaminase (AST), total bile acid (TBA), total bilirubin (T-BIL), direct bilirubin (d-BIL), glucose (GLC), triglycerides (TG), total cholesterol (TC), high-density lipoprotein (HDL), low-density lipoprotein (LDL), urea nitrogen (BUN), and creatinine (CREA) in serum (see Tables S1 and S2 in the supplemental material). Histopathology and clinical biochemistry analyses were conducted by the Wuhan Servicebio Technology Co., Ltd.

### Quantification of tryptophan metabolites.

Quantification of tryptophan metabolites in fecal, serum, liver, and colon tissues was performed by multiple-reaction monitoring (MRM) using an ultrahigh-performance liquid chromatograph (Agilent 1290) coupled with a model 6460 triple-quadrupole mass spectrometer (UHPLC-QQQ MS; Agilent Technologies, Inc.). The procedures of sample preparation and tryptophan metabolite measurements were carried out as described previously (55), with some improvements, as mentioned in Text S1.

10.1128/mSystems.00985-20.1 TEXT S1 Supplemental materials and methods. Download Text S1, DOCX file, 0.02 MB. Copyright © 2021 Shi et al. 2021 Shi et al. https://creativecommons.org/licenses/by/4.0/ This content is distributed under the terms of the Creative Commons Attribution 4.0 International license.

### Quantification of SCFAs and LCFAs.

Targeted analyses of short-chain fatty acids (SCFAs) in the cecal contents and of long-chain fatty acids (LCFAs) in the livers and sera of mice were performed on a Shimadzu 2010 Plus gas chromatograph (GC) MS (Shimadzu Scientific Instruments) equipped with a flame ionization detector (FID) and a CP-FFAP CB capillary GC column (SCFAs, 25 m by 0.32 mm, 0.3 μm; LCFAs, 10 m by 0.1 mm, 0.1 μm; Agilent Technology). 2,2-Dimethylbutyric acid and C17:0 were used as internal standards for quantification of SCFAs and LCFAs, respectively. The procedures of sample preparation and fatty acid measurements were as described in references 56 and 57 and in Text S1.

### NMR-based metabolomics.

^1^H NMR spectra of all the biological samples were recorded at 298 K on a Bruker Avance III 600-MHz spectrometer equipped with a Bruker inverse cryogenic probe (Bruker BioSpin). The sample preparation, NMR spectral acquisition and processing, and multivariate data analysis were performed as described previously (58) and in Text S1.

### Gut microbiota analysis.

For 16S rRNA gene sequencing analysis, the total DNA of cecal contents (∼100 mg) was extracted and a 16S rRNA gene amplicon sequence library was prepared as described in the protocol for 16S metagenomic sequencing library preparation (Illumina, USA). The V3-V4 region of 16S rRNA gene was amplified using a KAPA HiFi HotStart PCR kit (KAPA Biosystems, USA). Paired-end sequencing (2× 300 bp) was performed using an Illumina MiSeq platform from Shanghai Majorbio Bio-pharm Technology Co., Ltd. The preparation of the 16S rRNA gene amplicon sequence library, statistical analysis, and data manipulation are described in Text S1.

For metagenomics analysis, total genomic DNA was extracted from cecal contents (∼100 mg). DNA extract was fragmented to an average size of about 300 bp using a Covaris M220 apparatus (Gene Company Limited, China) for paired-end library construction. A paired-end library was constructed using a NEXTflex Rapid DNA-Seq kit (Bioo Scientific, Austin, TX, USA). Paired-end sequencing was performed on an Illumina HiSeq 4000 sequencer (Illumina Inc., San Diego, CA, USA) at Majorbio Bio-Pharm Technology Co., Ltd. (Shanghai, China) using HiSeq X reagent kits according to the manufacturer’s instructions. The quality filtering, assembling, gene prediction, and annotation of metagenomics data are described in Text S1.

### qPCR and Western blot analysis.

Total RNA was extracted from about 100 mg of frozen liver, ileum, and proximal colon tissues with RNAiso Plus reagent (TaKaRa). One microgram of RNA was used to synthesize cDNA with a PrimeScript RT reagent kit (TaKaRa). Using SYBR master mix, quantitative real-time PCR (qPCR) was conducted with a real-time PCR system (ABI StepOne; Applied Biosystems Co., Ltd., China). With glyceraldehyde-3-phosphate dehydrogenase (*Gapdh*) as a reference, qPCR conditions were set to 40 cycles of 95°C for 20 s, 95°C for 30 s, and 60°C for 30 s. The ΔΔ*C_T_* method, where *C_T_* is the threshold cycle, was used in the reaction analysis (29).

Liver and colon tissues were lysed in radioimmunoprecipitation assay (RIPA) buffer containing protease inhibitors (Beyotime Biotechnology). Protein concentration was determined with a bicinchoninic acid (BCA) protein assay kit (Sangon Biotech), and 50 μg of total protein was separated by 10% sodium dodecyl sulfate polyacrylamide gel electrophoresis and transferred onto polyvinylidene difluoride (PVDF) membranes. Primary antibodies for β-actin and AHR (both from Proteintech Group) and appropriate horseradish peroxidase (HRP)-conjugated secondary antibodies were used. The blots were analyzed using the enhanced-chemiluminescence (ECL) HRP substrate (Millipore) and ChemiDoc Imager (Bio-Rad). Protein levels were referenced to β-actin levels.

### ELISA analysis.

The concentrations of liver and colonic AHR, hepatic triglyceride (TG), serum lipopolysaccharide (LPS) and CD14, and proinflammatory cytokines (including IL-1β, IL-6, and tumor necrosis factor alpha [TNF-α]) in the sera and livers of mice were measured using ELISA kits (Shanghai Huyu Biotechnology Co., Ltd.) according to the manufacturer’s instructions.

### Statistical analysis.

All experimental values are presented as means ± standard deviations (SD). OriginLab software (Origin 2017) and GraphPad Prism software (Graph-Pad 7.0) were used for data analysis and graphical illustrations. All data between different groups were statistically analyzed by the Mann-Whitney U test or the Kruskal-Wallis analysis of variance (ANOVA) test. *P* values of <0.05 were considered significant.

### Data availability.

Sequencing data for the metagenomic sequences have been deposited in the SRA database under project no. PRJNA631099.
