# Supplementary figures and images for: Impaired Intestinal Akkermansia muciniphila and Aryl Hydrocarbon Receptor Ligands Contribute to Nonalcoholic Fatty Liver Disease in Mice
Source: mSystems. 2021 Feb 23;6(1):e00985-20. doi: 10.1128/mSystems.00985-20 (PMC8573958; doi:10.1128/mSystems.00985-20)

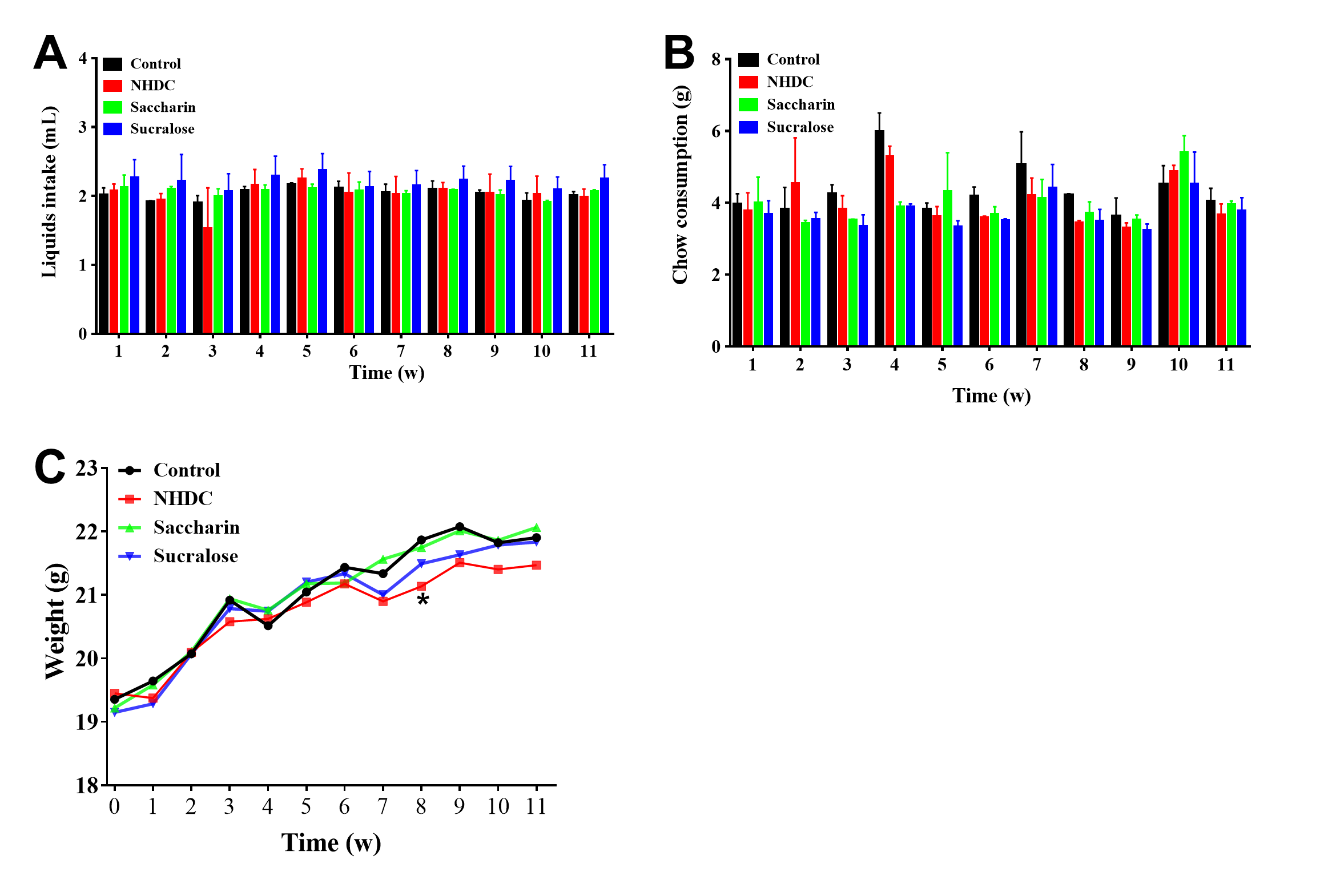

Supplement: FIG S1 [file msystems.00985-20-sf001.tif]

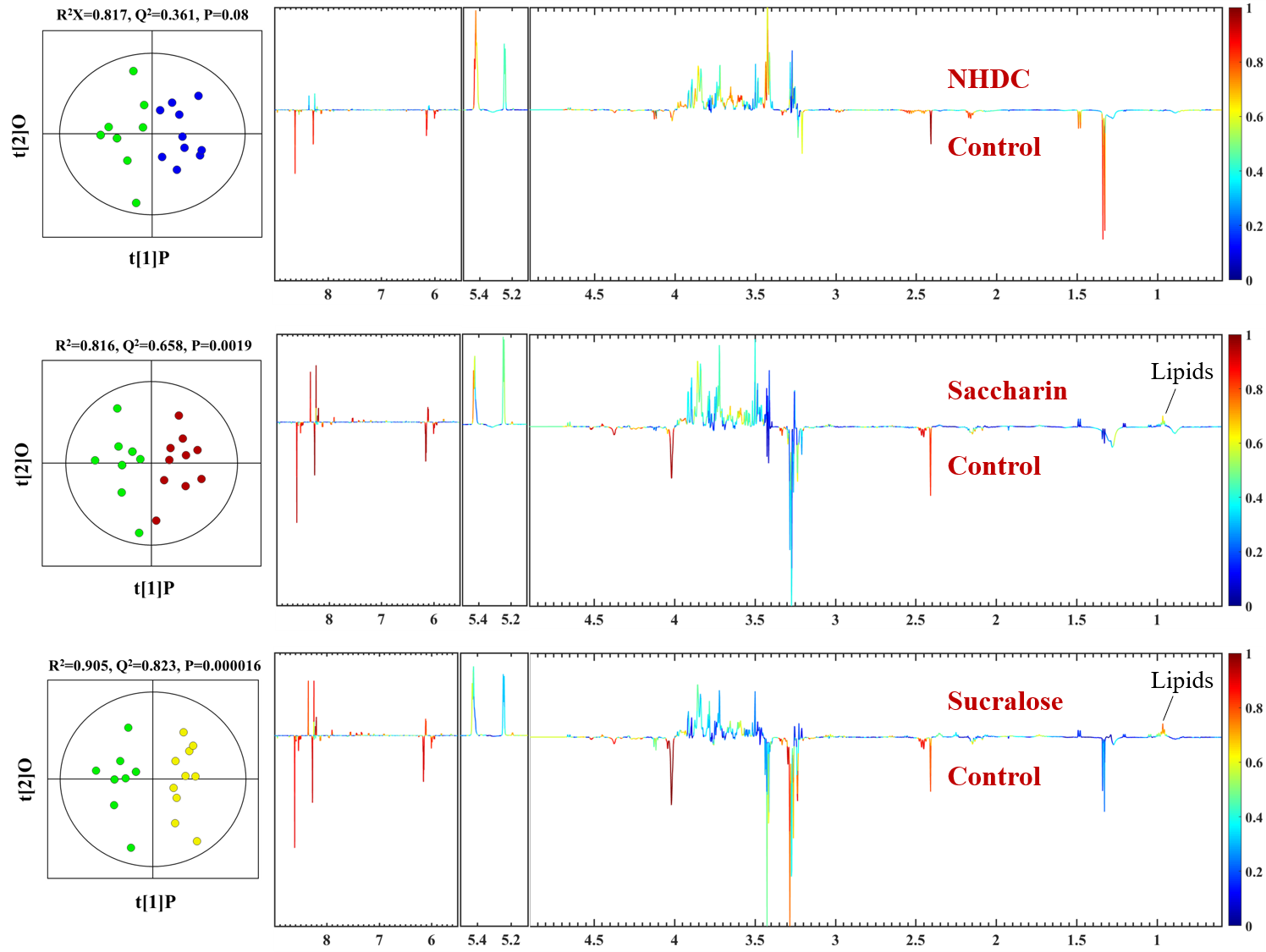

Supplement: FIG S2 [file msystems.00985-20-sf002.tif]

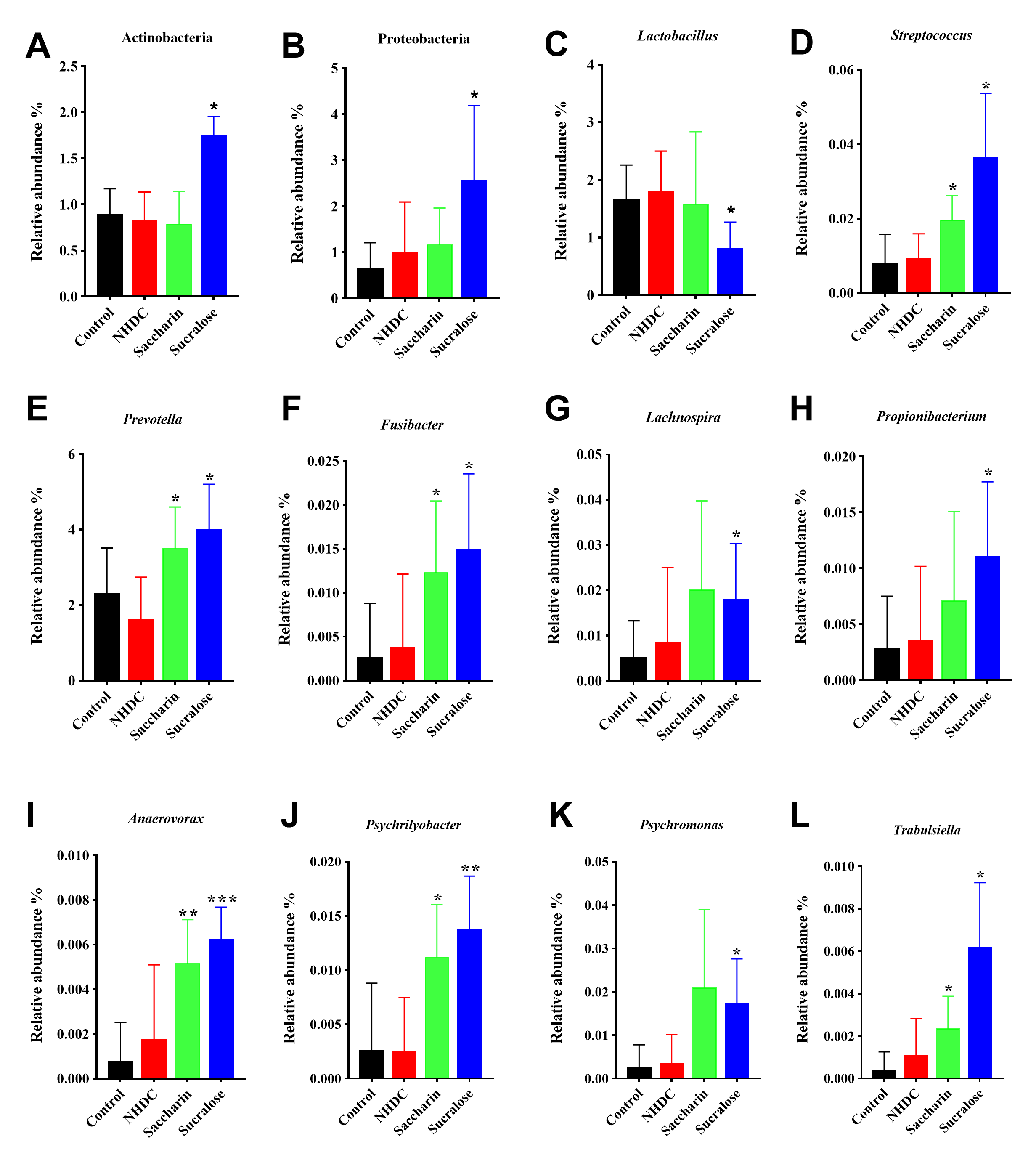

Supplement: FIG S3 [file msystems.00985-20-sf003.tif]

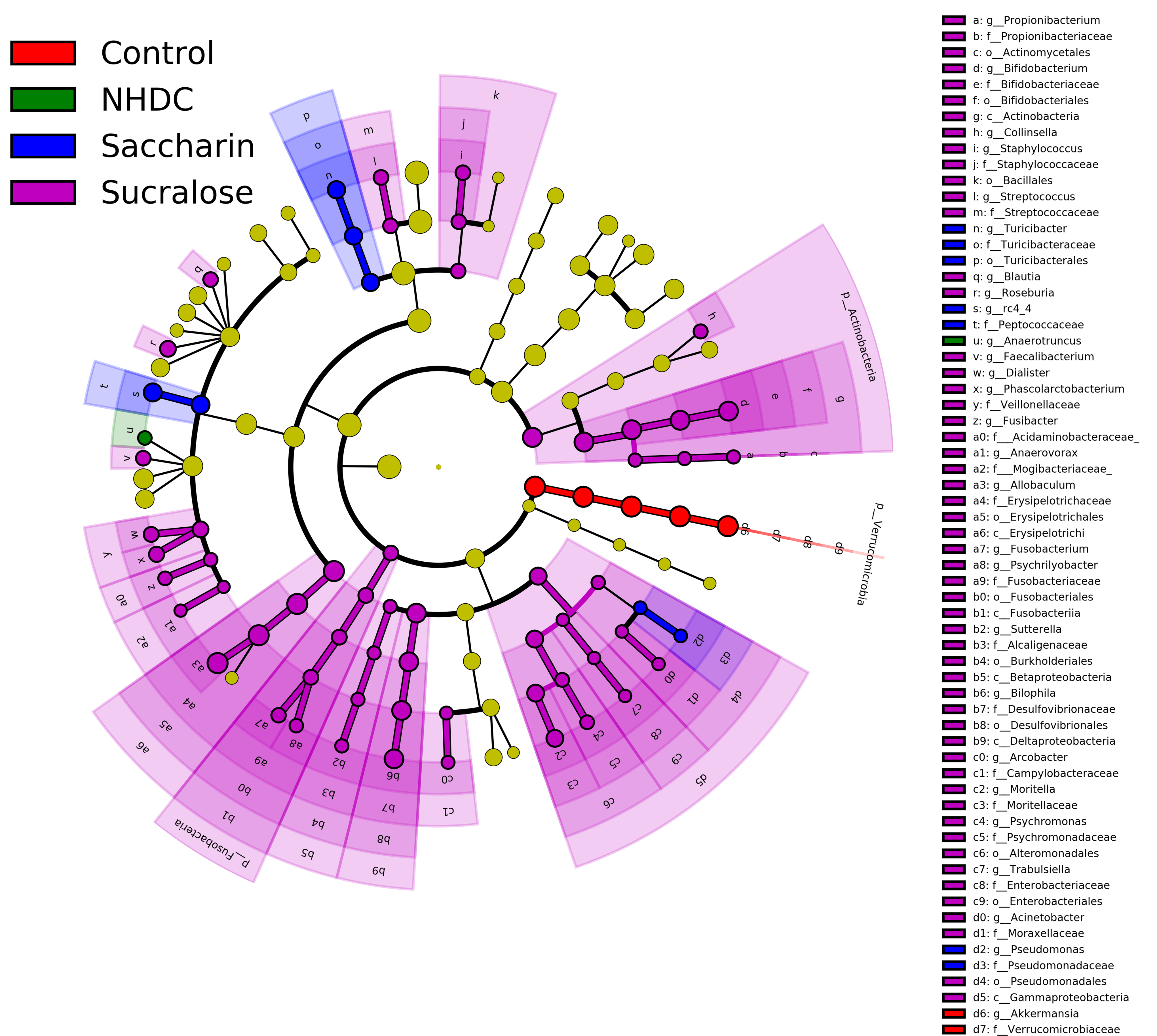

Supplement: FIG S4 [file msystems.00985-20-sf004.tif]

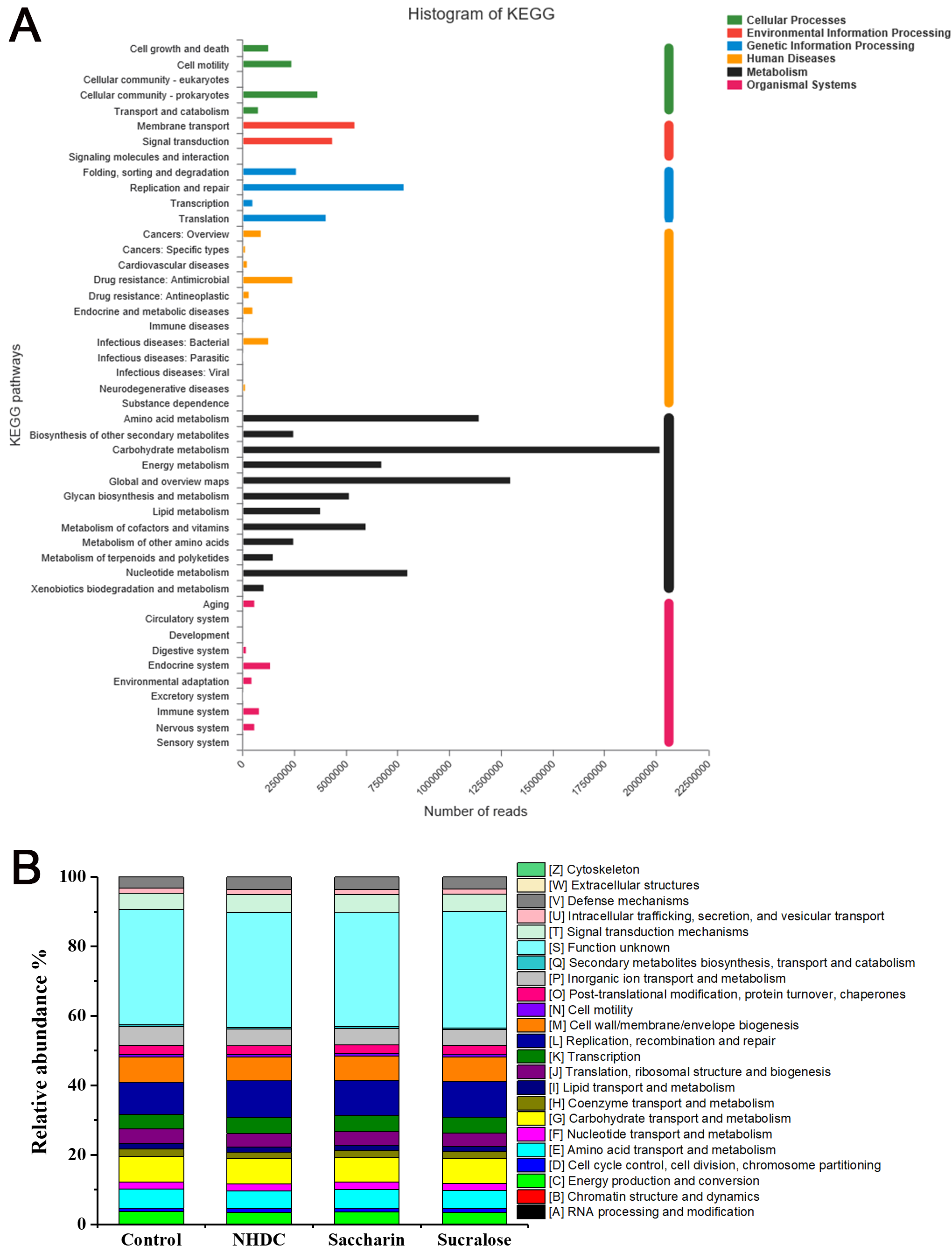

Supplement: FIG S5 [file msystems.00985-20-sf005.tif]

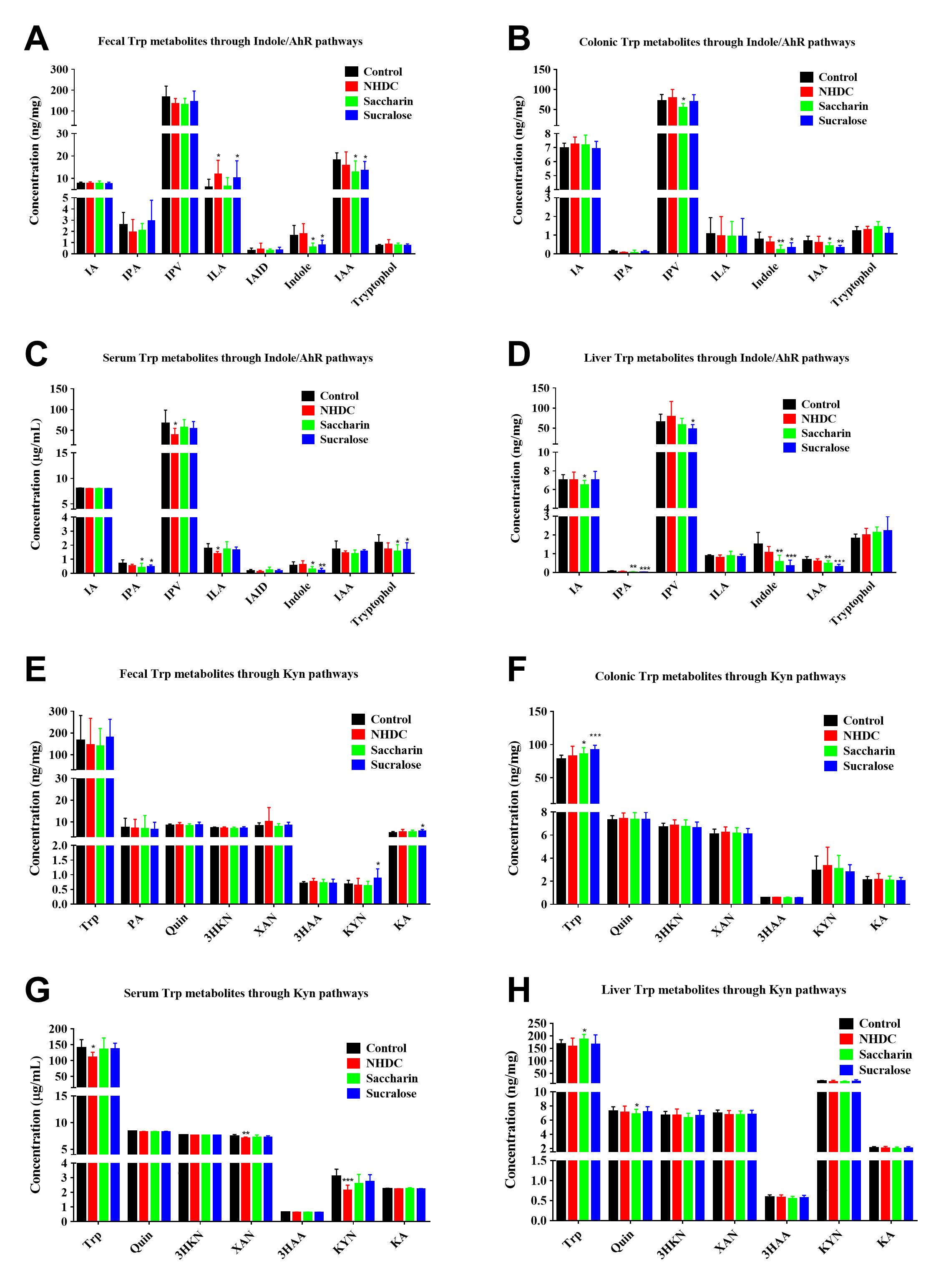

Supplement: FIG S6 [file msystems.00985-20-sf006.tif]

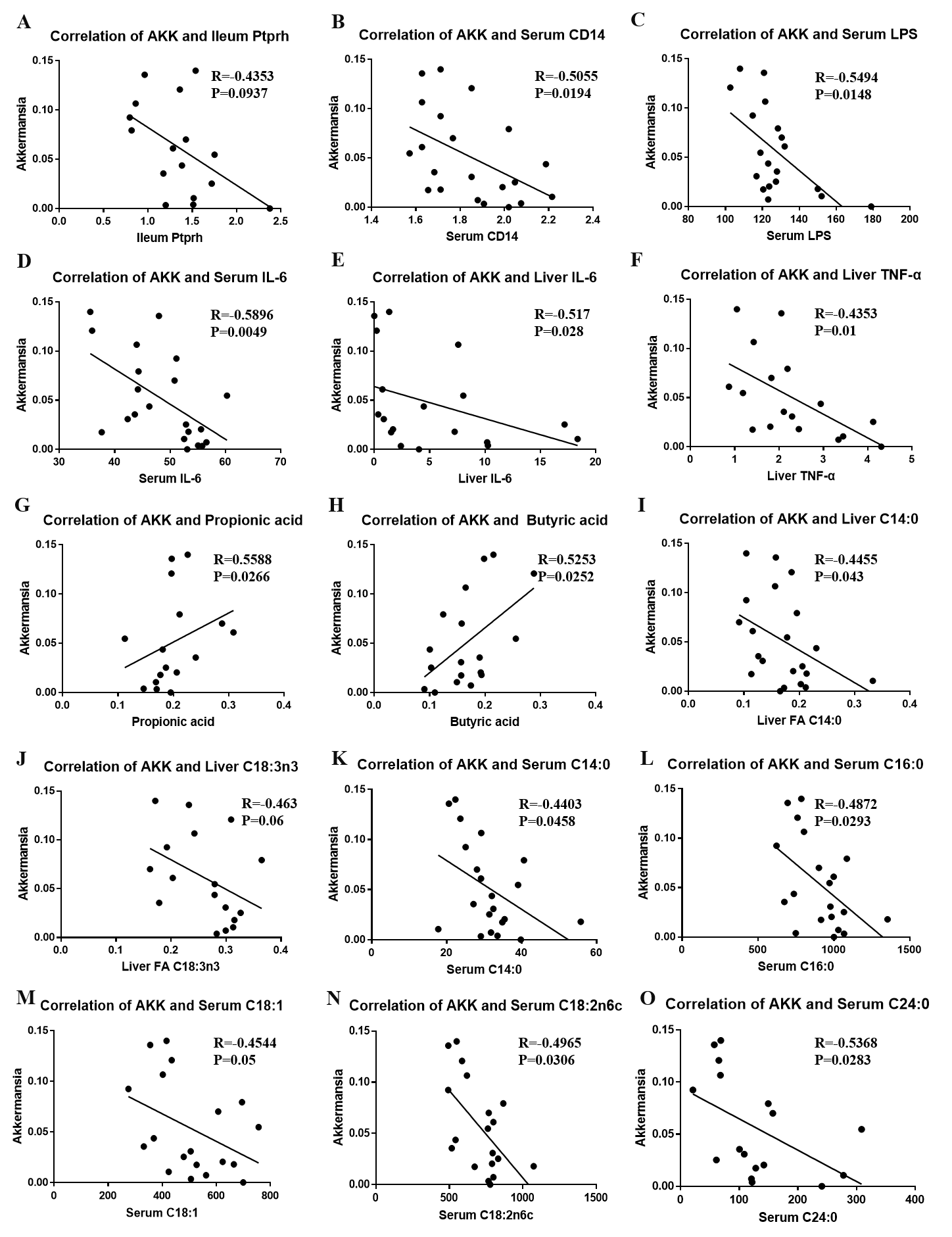

Supplement: FIG S7 [file msystems.00985-20-sf007.tif]
