# Supplementary material for: Impaired Intestinal Akkermansia muciniphila and Aryl Hydrocarbon Receptor Ligands Contribute to Nonalcoholic Fatty Liver Disease in Mice
Source: mSystems. 2021 Feb 23;6(1):e00985-20. doi: 10.1128/mSystems.00985-20 (PMC8573958; doi:10.1128/mSystems.00985-20)
Supplement: TABLE S1 [file msystems.00985-20-st001.docx]

| Parameters | Control | NHDC | Saccharin | Sucralose |
| --- | --- | --- | --- | --- |
| ALT (U/L) | 13.76±4.41 | 13.15±3.78 | 20.77±4.61^*^ | 20.12±4.42^*^ |
| ALP (U/L) | 133.55±6.73 | 147.42±13.89 | 151.38±11.85^*^ | 158.74±13.64^**^ |
| AST (U/L) | 99.98±13.91 | 98.23±13.45 | 97.52±22.18 | 107.10±25.29 |
| TBA (μmol/L) | 7.14±1.93 | 7.63±1.45 | 7.09±1.55 | 10.51±1.90^*^ |
| T-BIL (μmol/L) | 29.21±1.66 | 28.38±0.72 | 28.94±0.98 | 28.38±1.18 |
| D-BIL (μmol/L) | 18.88±0.93 | 17.85±0.68 | 17.85±0.87 | 17.9±0.82 |
| GLC (mmol/L) | 6.77±0.57 | 6.64±0.31 | 6.20±0.27 | 6.80±0.50 |
| TG (mmol/L) | 0.53±0.08 | 0.57±0.13 | 0.59±0.14 | 0.63±0.04^*^ |
| TC (mmol/L) | 1.38±0.05 | 1.37±0.06 | 1.32±0.09 | 1.37±0.06 |
| HDL (mmol/L) | 0.32±0.04 | 0.31±0.05 | 0.24±0.06^*^ | 0.30±0.03 |
| LDL (mmol/L) | 0.44±0.03 | 0.42±0.04 | 0.38±0.06 | 0.38±0.11 |
| BUN (mg/dL) | 19.94±6.37 | 20.31±2.08 | 18.09±3.66 | 18.88±1.74 |
| CREA (μmol/L) | 8.76±5.22 | 9.89±2.91 | 8.33±4.90 | 9.20±5.14 |

Values are presented as mean ± S.D. (n=6). * *P* <0.05 and ** *P* <0.01 indicate statistically significant differences when compared to control group.
