# Supplementary material for: Impaired Intestinal Akkermansia muciniphila and Aryl Hydrocarbon Receptor Ligands Contribute to Nonalcoholic Fatty Liver Disease in Mice
Source: mSystems. 2021 Feb 23;6(1):e00985-20. doi: 10.1128/mSystems.00985-20 (PMC8573958; doi:10.1128/mSystems.00985-20)
Supplement: TABLE S2 [file msystems.00985-20-st002.docx]

| Parameters | Control | Sucralose | Sucralose/  Metformin | Sucralose/FOS |
| --- | --- | --- | --- | --- |
| ALT (U/L) | 25.52±2.03 | 30.26±3.87* | 24.61±1.89^#^ | 23.95±2.71^##^ |
| ALP (U/L) | 147.68±17.37 | 167.71±13.99* | 122.66±26.3^##^ | 151.87±11.88^#^ |
| AST (U/L) | 80.62±9.68 | 91.65±5.45 | 82.36±4.21 | 83.82±8.25 |
| T-BIL (μmol/L) | 24.72±4.67 | 24.59±4.35 | 23.64±4.29 | 20.79±2.99 |
| D-BIL (μmol/L) | 13.33±1.87 | 15.44±6.35 | 15.01±1.60 | 15.28±4.42 |
| GLC (mmol/L) | 7.19±0.83 | 7.49±2.61 | 5.94±0.74^#^ | 6.16±0.70 |
| TG (mmol/L) | 0.59±0.01 | 0.64±0.05* | 0.61±0.03 | 0.59±0.02^#^ |
| TC (mmol/L) | 1.28±0.15 | 1.48±0.19* | 1.23±0.12^#^ | 1.29±0.11^#^ |
| HDL (mmol/L) | 0.17±0.03 | 0.21±0.05 | 0.15±0.02 | 0.19±0.02 |
| LDL (mmol/L) | 0.64±0.06 | 0.81±0.16* | 0.66±0.09 | 0.72±0.08 |
| BUN (mg/dL) | 23.94±2.89 | 22.92±3.19 | 18.45±2.30^#^ | 16.59±2.20^#^ |
| CREA (μmol/L) | 13.48±5.31 | 11.73±3.17 | 10.84±3.67 | 8.46±2.29 |

Values are presented as mean ± S.D. (n=6). * *P* <0.05 and ** *P* <0.01 indicate statistically significant differences when compared to control group. ^#^ *P* <0.05 and ^##^ *P* <0.01 indicate statistically significant differences when compared to sucralose treatment group.
