# Supplementary material for: Impaired Intestinal Akkermansia muciniphila and Aryl Hydrocarbon Receptor Ligands Contribute to Nonalcoholic Fatty Liver Disease in Mice
Source: mSystems. 2021 Feb 23;6(1):e00985-20. doi: 10.1128/mSystems.00985-20 (PMC8573958; doi:10.1128/mSystems.00985-20)
Supplement: TEXT S1 [file msystems.00985-20-s0001.docx]

**Supplemental materials and methods**

**Chemicals.** NHDC, saccharin and sucralose were obtained from Sigma-Aldrich Chemical Co., Ltd. (St, Louis, MO, USA). Metformin and oligosaccharides (FOS) were purchased from Sigma-Aldrich Chemical Co., Ltd. (St, Louis, MO, USA). 3-(trimethylsilyl) propionic-2,2,3,3-d4 acid sodium salt (TSP-d4, 98% D) and D_2_O (99.9% D) were purchased from Sigma-Aldrich Chemical Co., Ltd. (St, Louis, MO, USA). Heparin sodium, sodium chloride, diethyl ether, isoflurane anesthesia, hydrochloric acid (HCl), K_2_HPO_4_·3H_2_O, and NaH_2_PO_4_·2H_2_O were obtained from Sinopharm Chemical Reagent Co. Ltd. (Shanghai, China). HPLC-grade water, formic acid (FA), acetonitrile (MeCN) and methanol (MeOH) were purchased from ThermoFisher Scientific Co., Ltd. (Shanghai, China). 3,5-Ditertbutyl-4-hydroxytoluene (BHT) were obtained from Supelco (Bellefonte, PA, USA), and standards of tryptophan metabolites and fatty acids were obtained from Sigma-Aldrich Chemical Co., Ltd. (St, Louis, MO, USA).

**Quantification of Tryptophan Metabolites.** Tryptophan metabolites extraction was performed from feces, liver and colon tissues (~10 mg). Samples mixed with internal standard (10 μL d5-TRP) were respectively homogenized with 400 μL cooled methanol and 50 μL acetonitrile: water solution (1:1 v/v) containing 0.1% formic acid using the Qiagen Tissue-Lyser (Retsch GmBH, Germany) at 20 Hz for 90 s. After extraction for two times, the combined supernatants were collected and evaporated into dryness following centrifugation. Serum sample (10 μL) was uniformly mixed with 10 μL of internal standard (d5-TRP), 150 μL cooled methanol and 10 μL acetonitrile: water solution (1:1 v/v) containing 0.1% formic acid. After centrifugation for 20 min (4 °C), the supernatants were collected and lyophilized for removing methanol in vacuum. Dried extracts were reconstituted in 100 μL of acetonitrile: water solution (1:1 v/v) containing 0.1% formic acid. Qualitative and quantitative analyses of tryptophan metabolites were performed using an ultrahigh performance liquid chromatography (Agilent 1290) coupled with a 6460 triple quadrupole mass spectrometry (UHPLC-QQQ-MS, Agilent Technologies, Inc.). The precursor ions of tryptophan metabolites were pre-scanned through multiple reaction monitoring (MRM) of all sample mixtures and the structures were identified through MS/MS spectra. Quantification of tryptophan metabolites was performed using calibration curves based on MRM and the ratios of the integrated peak areas of tryptophan metabolites and internal standards.

**Quantification of Short Chain Fatty Acids and Long Chain Fatty Acids.** The short chain fatty acids (SCFAs) extraction was performed from cecal contents (~20 mg). Samples and internal standard (10 μL, 2,2-dimethylbutyric acid) was mixed with 400 μL 1 N HCl. After vortexing and three continuous freeze−thaw cycle, the mixture was extracted with 400 μL diethyl ether. Following centrifugation, 200 μL supernatants were collected and analyzed on a Shimadzu 2010 Plus GC-MS spectrometer (Shimadzu Scientific Instruments) equipped with a flame ionization detector (FID) and a CP-FFAP CB capillary GC column (25 m × 0.32 mm, 0.3 μm, Agilent Technology). Helium was used as the carrier gas and the injection volume was 1 μL. The programmed column temperature was as follows: the temperature of injection port and detector was set at 250 °C, the temperature of oven was increased from 100 °C to 200 °C at a rate of 10 °C per min. SCFAs were identified by comparing retention time with a mixture of 9 SCFAs standards. Quantification of SCFAs was subsequently conducted using calibration curves of internal standards (2,2-dimethylbutyric acid) and calculation as nanomoles of SCFA per gram of cecal contents.

The methylesterification of long chain fatty acids (LCFAs) was conducted from liver and serum of mice. Liver tissue (~10 mg) was mixed with 500 μL methanol and a 5-mm tungsten carbide bead (Qiagen, Germany). After vortexing, the mixture was homogenized using a TissueLyser (Qiagen, Germany). 100 μL of liver homogenate and 20 μL internal standards (1 mg/mL C17:0 methyl ester, 0.5 mg/mL C23:0 fatty acid, 2 mg/mL BHT) were mixed with 1 mL methanol: hexane solution (4:1 v/v). The methylation reaction was initiated by addition of acetyl chloride (100 μL) and kept for 24 h in the dark at 25 °C, neutralized with 2.5 mL K_2_CO_3_ solution on ice. The methylated fatty acid solution was extracted four times with 200 μL hexane. After centrifugation, the lipid phase fraction was combined and evaporated to dryness at room temperature in a fume hood. In addition, serum (50 μL) was dissolved in 1 mL methanol/hexane solution (4:1, v/v,) containing butylated hydroxytoluene and internal standard solution (20 μL) including C17:0 (1 mg/mL) and C23:0 (0.5 mg/mL). The methylation reaction was initiated by adding acetyl chloride (100 μL) at 25 °C in the dark kept for 24 h and stopped by addition of 2.5 mL 6% K_2_CO_3_ solution on ice. The methylated long chain fatty acids solution was extracted with 200 μL hexane for three times. The combined supernatants were evaporated to dryness at room temperature in a fume hood. The methylated long chain fatty acids of liver and serum were dissolved in hexane (100 μL) and analyzed on a Shimadzu 2010 Plus GC-MS spectrometer (Shimadzu Scientific Instruments, Columbia, MD) equipped with a flame ionization detector (FID) and a DB-225 capillary GC column (10 m × 0.1 mm, 0.1 μm, Agilent Technology). Helium was used as the carrier gas and the injection volume was 1 μL. Temperature of injection port and detector was set at 230 °C. The programmed column temperature was as follows: the temperature of oven was increased from 55 °C to 205 °C at a rate of 25 °C per min, kept at 205 °C for 3 min and then increased to 225 °C at a rate of 10 °C per min. The temperature was then kept at 225 °C for further 3min. Methylated long chain fatty acids were identified by comparing retention time with a mixture of 37 fatty acid standards. The long chain fatty acids composition was subsequently quantified using calibration curves of internal standards (C17:0 and C23:0) and calculated as nanomoles of long chain fatty acids per gram of liver.

**NMR-Based Metabolomics.** About 50 mg liver tissues were extracted three times with cooled methanol/water extraction solution (2/1, v/v) using the Qiagen Tissue-Lyser (Retsch GmBH, Germany) at 20 Hz for 90 s. After collection of all three extracts, the combined supernatants were lyophilized for removing methanol by vacuum Freeze Drier. The obtained powder was reconstituted in 600 μL phosphate buffer (0.1 M, K_2_HPO4:NaH_2_PO4 = 4:1, pH ≈ 7.4) containing 50% D_2_O and 0.001% TSP as an internal reference. Typical one-dimensional ^1^H NMR spectra were acquired for liver extracts at 298 K on a Bruker Avance III 600 MHz spectrometer (Bruker BioSpin, Germany) equipped with a Bruker inverse detection cryogenic probe. Standard NOESY pulse sequence (recycle delay-90°-t_1_-90°-t_m_-90°-acquisition) was used for liver extracts with parameters as recycle delay time of 2.0 s, t_1_ of 3.0 μs, and mixing time (t_m_) of 80 ms. For NMR signals identification, several two-dimensional (2D) NMR spectra including ^1^H−^1^H COSY, ^1^H−^1^H TOCSY, ^1^H−^13^C HSQC, and ^1^H−^13^C HMBC were recorded for liver samples. All ^1^H NMR spectra phase and baseline were manually corrected and referenced internally to the TSP peak at 0.00 ppm for liver extracts using Topspin 3.6 (V3.1, Bruker Biospin, Germany). The spectra region δ 0.5-δ 9.6 ppm with removal of water signals were integrated with an equal width of 0.002 ppm (1.2 Hz) for all the samples using the MestRenova 9.0. After integration, the wet weight normalization method was performed for each bucketed region of liver extracts. Multivariate data statistical analysis was conducted using SIMCA-P+ 13.0 (Umetrics, Sweden). In brief, principal component analysis (PCA) was firstly used to check the information on group separation and potential outliers. Orthogonal projection to latent structures with discriminant analysis (OPLS-DA) was subsequently employed using NMR data scaled to unit variance. All the models were further assessed by a 7-fold cross-validation with CV-ANOVA (*p* < 0.05). For extracting and interpreting the results of OPLS-DA model, the color-coded loading plots were conducted to extract or discriminant the significantly changed metabolites after back-transformation of the integrated NMR data using Matlab script (V7.8, MA).

**Gut microbiota analysis.** For 16S rRNA gene sequencing analysis, total DNA of cecal contents (~100 mg) was extracted using the E.Z.N.A.® soil DNA Kit (Omega Bio-tek, Norcross, GA, U.S.), according to the manufacturer’s protocol. The 16S rRNA gene amplicon sequence library was prepared as described in the protocol of 16S Metagenomic Sequencing Library Preparation (Illumina, United States). Briefly, the V3-V4 region of 16S rRNA gene was ampliﬁed using a KAPA HiFi HotStart PCR Kit (KAPA Biosystem, USA). Dual-index barcodes were added to the amplicon target by the index PCR using a Nextera® Index Kit (Illumina, USA). Amplicons were puriﬁed using AMPure XP beads and quantiﬁed using a KAPA library quantitative kit (KAPA Biosystem, USA). Equimolar amounts of puriﬁed amplicons were pooled and paired-end sequencing (2 × 300 bp) were performed using an Illumina MiSeq platform by Shanghai Majorbio Bio-pharm Technology Co., Ltd. After demultiplexing, the resulting sequences were merged with FLASH ( v1.2.11) and quality filtered with fastp (0.19.6). Then the high-quality sequences were de-noised using Deblur plugin in the Qiime2 (version 2020.2) pipeline with ecommended parameters, which obtained single nucleotide resolution based on error profiles within samples. Deblur denoised sequences are usually called amplicon sequence variants (ASVs). In this study, these denoised sequences were assigned to bacterial features, which are synonymous to ASVs. To minimize the effects of sequencing depth on alpha and beta diversity measure, the number of sequence from each sample was rarefied to 20000, which still yielded an average Good’s coverage of 97.90%. The taxonomy of these features was performed using the Vsearch consensus taxonomy classifier implemented in Qiime2 and the SILVA 16S rRNA database (version 138). Most statistical analysis and data manipulation were conducted with vegan, reshape2, and ggplot2 packages using R software. The α-diversity (Number of Features) was calculated with vegan and fossil using R software. Differences in community composition across samples (β-diversity) were represented by Bray-Curtis dissimilarities calculated from the feature table, and principal co-ordinates analysis (PCoA) was used to display and compare the patterns of microbial communities.

For metagenomics analysis, total genomic DNA was extracted from cecal contents (~100 mg). Concentration and purity of extracted DNA was determined with TBS-380 and NanoDrop2000, respectively. DNA extract was fragmented to an average size of about 300 bp using Covaris M220 (Gene Company Limited, China) for paired-end library construction. Paired-end library was constructed using NEXTFLEX Rapid DNA-Seq (Bioo Scientific, Austin, USA). Adapters containing the full complement of sequencing primer hybridization sites were ligated to the blunt-end of fragments. Paired-end sequencing was performed on Illumina Hiseq 4000 (Illumina Inc., San Diego, CA, USA) at Majorbio Bio-Pharm Technology Co., Ltd. (Shanghai, China) using HiSeq X Reagent Kits according to the manufacturer’s instructions. Adapter sequence were stripped from the 3’ and 5’ end of paired end Illumina reads using SeqPrep (https://github.com/jstjohn/SeqPrep). Low-quality reads (length<50 bp or with a quality value <20 or having N bases) were removed by Sickle (https://github.com/najoshi/sickle). Reads were aligned to the musculus genome by NCBI and any hit associated with the reads and their mated reads were removed. Metagenomics data were assembled using MEGAHIT. Contigs with the length being or over 300 bp were selected as the final assembling result, and then the contigs were used for further gene prediction and annotation. Open reading frames (ORFs) from each assembled contig were predicted using MetaGene. All predicted genes with a 95 % sequence identity (90% coverage) were clustered using CD-HIT, the longest sequences from each cluster were selected as representative sequences to construct non-redundant gene catalog. Reads after quality control were mapped to the representative sequences with 95% identity using SOAPaligner, and gene abundance in each sample were evaluated. Representative sequences of non-redundant gene catalog were aligned to NCBI NR database with e-value cutoff of 1e^-5^ using BLASTP (Version 2.2.28+) for taxonomic annotations. Cluster of orthologous groups of proteins (COG) annotation for the representative sequences was performed using BLASTP against eggNOG database with an e-value cutoff of 1e^-5^. KEGG database was also used for gene annotation using BLASTP (Version 2.2.28+).
